# Tumour response to hypoxia: understanding the hypoxic tumour microenvironment to improve treatment outcome in solid tumours

**DOI:** 10.3389/fonc.2024.1331355

**Published:** 2024-01-30

**Authors:** Kamilla JA. Bigos, Conrado G. Quiles, Sapna Lunj, Danielle J. Smith, Mechthild Krause, Esther GC. Troost, Catharine M. West, Peter Hoskin, Ananya Choudhury

**Affiliations:** ^1^ Division of Cancer Sciences, University of Manchester, Manchester, United Kingdom; ^2^ German Cancer Consortium (DKTK), partner site Dresden and German Cancer Research Center (DKFZ), Heidelberg, Germany; ^3^ Department of Radiation Oncology, Faculty of Medicine and University Hospital Carl Gustav Carus, Technische Universität Dresden, Dresden, Germany; ^4^ OncoRay – National Center for Radiation Research in Oncology, Faculty of Medicine and University Hospital Carl Gustav Carus, Technische Universität Dresden, Helmholtz-Zentrum Dresden - Rossendorf, Dresden, Germany; ^5^ Translational Radiooncology and Clinical Radiotherapy, Helmholtz-Zentrum Dresden - Rossendorf, Dresden, Germany; ^6^ Translational Radiation Oncology, National Center for Tumor Diseases (NCT), Partner Site Dresden, Dresden, Germany; ^7^ Translational Radiooncology and Clinical Radiotherapy and Image-guided High Precision Radiotherapy, German Cancer Research Center (DKFZ), Heidelberg, Germany; ^8^ Faculty of Medicine and University Hospital Carl Gustav Carus, Technische Universität Dresden, Dresden, Germany; ^9^ Translational Radiooncology and Clinical Radiotherapy and Image-guided High Precision Radiotherapy, Helmholtz Association / Helmholtz-Zentrum Dresden - Rossendorf (HZDR), Dresden, Germany; ^10^ School of Medicine, Technische Universitat Dresden, Dresden, Germany; ^11^ Department of Radiotherapy and Radiation Oncology, Faculty of Medicine and University Hospital Carl Gustav Carus, Technische Universität Dresden, Dresden, Germany; ^12^ Institute of Radiooncology – OncoRay, Helmholtz-Zentrum Dresden-Rossendorf, Rossendorf, Germany; ^13^ Division of Cancer Sciences, University of Manchester, Manchester Academic Health Science Centre, Christie Hospital, Manchester, United Kingdom; ^14^ Mount Vernon Cancer Centre, Northwood, United Kingdom; ^15^ Christie Hospital NHS Foundation Trust, Manchester, Germany

**Keywords:** hypoxia, tumour microenvironment, extracellular matrix, immune cells, cancer associated fibroblasts

## Abstract

Hypoxia is a common feature of solid tumours affecting their biology and response to therapy. One of the main transcription factors activated by hypoxia is hypoxia-inducible factor (HIF), which regulates the expression of genes involved in various aspects of tumourigenesis including proliferative capacity, angiogenesis, immune evasion, metabolic reprogramming, extracellular matrix (ECM) remodelling, and cell migration. This can negatively impact patient outcomes by inducing therapeutic resistance. The importance of hypoxia is clearly demonstrated by continued research into finding clinically relevant hypoxia biomarkers, and hypoxia-targeting therapies. One of the problems is the lack of clinically applicable methods of hypoxia detection, and lack of standardisation. Additionally, a lot of the methods of detecting hypoxia do not take into consideration the complexity of the hypoxic tumour microenvironment (TME). Therefore, this needs further elucidation as approximately 50% of solid tumours are hypoxic. The ECM is important component of the hypoxic TME, and is developed by both cancer associated fibroblasts (CAFs) and tumour cells. However, it is important to distinguish the different roles to develop both biomarkers and novel compounds. Fibronectin (FN), collagen (COL) and hyaluronic acid (HA) are important components of the ECM that create ECM fibres. These fibres are crosslinked by specific enzymes including lysyl oxidase (LOX) which regulates the stiffness of tumours and induces fibrosis. This is partially regulated by HIFs. The review highlights the importance of understanding the role of matrix stiffness in different solid tumours as current data shows contradictory results on the impact on therapeutic resistance. The review also indicates that further research is needed into identifying different CAF subtypes and their exact roles; with some showing pro-tumorigenic capacity and others having anti-tumorigenic roles. This has made it difficult to fully elucidate the role of CAFs within the TME. However, it is clear that this is an important area of research that requires unravelling as current strategies to target CAFs have resulted in worsened prognosis. The role of immune cells within the tumour microenvironment is also discussed as hypoxia has been associated with modulating immune cells to create an anti-tumorigenic environment. Which has led to the development of immunotherapies including PD-L1. These hypoxia-induced changes can confer resistance to conventional therapies, such as chemotherapy, radiotherapy, and immunotherapy. This review summarizes the current knowledge on the impact of hypoxia on the TME and its implications for therapy resistance. It also discusses the potential of hypoxia biomarkers as prognostic and predictive indictors of treatment response, as well as the challenges and opportunities of targeting hypoxia in clinical trials.

## Hypoxia

Hypoxia is a state in which there is a lack of sufficient oxygen supply to tissues and organs. This has detrimental effects on cells as they require oxygen for their function. The physiological response to hypoxia is to induce cell death. In the context of cancer, overconsumption of oxygen leads to low levels of oxygen. However, tumour cells find mechanisms to adapt to these harsh conditions, enabling tumour cell survival. Hypoxia modulates tumour growth, invasion, and resistance to therapy induced by rapid tumour cell proliferation, abnormal tumour vasculature, high interstitial pressure, or low oxygen delivery. These combined features can enhance a tumours ability to metastasise (1, 2). Hypoxia can be described as chronic, acute or cycling within solid tumours. Cycling hypoxia is defined by tumours undergoing periodic exposure to hypoxia followed by reoxygenation, which is associated with enhancing common hallmarks of cancer (3, 4). This has a significant role in promoting resistance to both radiotherapy and chemotherapy (2). Schwarz et al., were the first to propose that hypoxia drives resistance to radiotherapy (5). Thomlinson and Gray also observed hypoxia in bronchial carcinomas. These tumours were found to grow in solid cords surrounded by stroma, cords larger than 180 microns, were associated with a necrotic centre due to insufficient oxygen supply (1). These landmark studies led researchers to explore the impact of hypoxia on anti-tumour response.

The hypoxic tumour microenvironment (TME) has been defined as a condition where the partial pressure of oxygen (pO_2_) is below 10 mmHg (6). Physoxia describes the maintenance of physiological levels of oxygen in tumours. It can differ dependent on tissue type and location, highlighting why hypoxia also varies between different tumour types (**Table 1**).

**Table 1:** Tissue and tumour physoxia and hypoxia expressed as partial pressure of O_2_ (pO_2_).

Tissue	Partial pressure of O_2_ (pO_2_) in tissue	Partial pressure of O_2_ (pO_2_) in tumour	Refs
Prostate	23-30	2.4-4.5	(7, 8)
Pancreas	9.3-92.7	0-5.3	(9, 10)
Lung	32-90 (11)	32-120	(12, 13)
Liver	30	6	(14)
Renal cell carcinoma	37.6	9.6	(15)
Cervix (nullipara)	9-14	42-48	(10, 16)
Breast	37-65	3-15	(10, 17)
Brain	24-27	13	(10)
Head and Neck	38-51.2	5-14.6	(12, 18–22)

## Hypoxia- inducible factor activation by hypoxia

HIF, a transcription factor responding to low oxygen levels, regulates gene expression to adapt tumour cell behaviour for survival (**Table 2**). It activates genes supporting hypoxia adaptation, like those linked to angiogenesis, erythropoiesis, glucose uptake, and anaerobic metabolism, while suppressing non-essential genes for survival (46).

**Table 2:** HIF molecule expression patterns and the pathways they target.

HIF molecule	Expression pattern	Target genes	Pathways regulated	Refs
HIF-1 (induced under severe hypoxia 0-2% O_2_)- acute hypoxia response	Endothelial cells, tumour cells, immune cells (neutrophils/macrophages), fibroblasts and cancer stem cells	VEGF, CA9, BNIP3, GLUT-1, VEGF, uPAR, IGF1, PDGF-B, NDUFA4L2, HGF, ITGA6, P4HA1, P4HA2, PLOD2, LOX, syndecan-4, α5-integrin MMP-2, MMP9, MMP15, NFκB	Angiogenesis, acid metabolism, cell death, glycolysis, angiogenesis, proteolytic pathway of invasion, cell-ECM interactions, enhanced invasion and migration, fibrosis-enhanced metastasis, promote survival and function of neutrophils, enhanced cell survival	(23–36)
HIF-2 (induced over severe conditions 2-5%)- long term hypoxia response	Endothelial cells, tumour cells , fibroblasts, immune cells (neutrophils, macrophages), cancer stem cells	VEGF (more potent response than HIF-1), GLUT-1, UPAR1, ITGA6 , MMP14	Associated with long-term hypoxic response, angiogenesis, glycolysis, proteolytic pathway of invasion, cell-ECM interactions, vessel integrity and tumour neovascularisation, promote survival and function of neutrophils, regulator of innate immunity, and metastasis	(25, 26, 28, 29, 37–41)
HIF-3	Endothelial cells, tumour cells		Apoptosis, tumorigenesis, negative regulator of HIF-1/HIF-2 in renal cell carcinoma	(42–45)

Cellular response to hypoxia induces intracellular signalling pathways regulated by HIF (2). HIF-1 is a heterodimer composed of an oxygen-sensitive subunit, HIF-1α, and HIF-1β which is constitutively expressed (47). Under physoxia, HIF-1α is degraded however, under hypoxia, HIF-1α is stabilised and forms a heterodimer with HIF-1β. This complex acts as a transcriptional activator that binds to DNA sequences called hypoxia response elements (HRE) in the promoters of target genes (14). HIF-2 is also a heterodimer with and oxygen-sensitive HIF-2α subunit, whilst sharing HIF-1β with HIF-1α as a constitutively expressed subunit (48). However, HIF-1 and HIF-2 have distinctive roles and this is why both are considered as separate therapeutic targets (**Table 2**). HIF-3 is the least characterised HIF and has multiple isoforms which are variants of HIF-3α. These isoforms have been identified with different tissue distribution and functional properties. HIF-3 does not have a transactivation domain like HIF-1/HIF-2 but instead contains a polypeptide that represses HRE-responsive gene expression (49).

## Hypoxia as a biomarker

The amount of hypoxia varies in solid tumours, with around 50% of tumours having high levels of hypoxia (50). Numerous studies show that most hypoxic tumours have the worse prognosis (51). Therefore, the importance of hypoxia as an adverse prognostic factor has led to interest in developing methods for its measurement and targeting (51).

In the 1960s, studies were published on the use of oxygen electrodes to measure hypoxia in cervical cancers (CC) (52). Oxygen electrodes (Eppendorf probes) or fibre optic probes (OxyLite) measure oxygen tension directly. The approach is based on inserting an electrode into an accessible tumour and measuring oxygen at multiple points within several tracks (51). Although this is considered the gold standard, it is invasive, operator-dependent and cannot account for heterogeneity (53). The result of heterogeneity is the overestimation of viable hypoxic cells as both areas with healthy cells and necrotic cells are measured (51, 54, 55). The key differences in the two methods are summarised by Griffiths & Robinson (53). The output of these methods is pO_2_ (**Table 1**). Fyles et al. (56) showed that oxygenation measured by oxygen electrodes was able to predict radiation response and survival in patients with CC. Nordsmark et al. (57) show that in advanced head and neck squamous cell carcinoma (HNSCC), patients with pO_2_ value of ≤2.5mmHg had a stronger predictive ability for radiation response. However, despite the ability to predict radiation response using this method, due to the limitations of the electrode systems, this led to their discontinuation (**Table 3**).

**Table 3:** Advantages and disadvantages of hypoxia detection methods.

Technique	Advantages	Disadvantages
**Oxygen electrode probes**	Well validated	Invasive
**Histopathology (e.g. necrosis)**	Extremely cheap	Hypoxia can appear without necrosis
	Easy to perform	No serial measurements
	Use of available diagnostic biopsies	
IHC* (GLUT-1, CA9, HIF-1)	Cheap	Not robust
	Easy to perform	Protein expression can be affected by external factors
	Suitable for large retrospective cohorts	Poor validation across studies
	Use of available diagnostic biopsies	No serial measurements
**Nitroimidazole markers**	Cheap	Needs to be administered 7-48h before biopsy
	Easy to perform	Staining variability across biopsies
	Validated	Do not detect acute hypoxic areas
**mRNA gene signatures**	Well validated (e.g breast, lung, head and neck cancers)	No consensus signatures
	Use of multiple genes allows for robust and replicable results	No serial measurements
	Use of available diagnostic biopsies	
**miRNA signatures**	Less vulnerable to degradation than mRNA	Not validated
	Use of multiple miRNAs allows for robust and replicable results	No serial measurements
	Use of available diagnostic biopsies	
**PET**	Allows serial measurements	Expensive
	Non-invasive	Complex image analysis

*IHC, immunohistochemistry.

Despite the limitations, the studies in oxygen measurement show that there is a clinical benefit to measuring hypoxia, which has led to continued research in the field including hypoxia targeted therapies (73). The methods include increasing oxygen delivery to tumours by breathing hyperbaric oxygen or carbogen; increasing vascular perfusion (e.g., nicotinamide); giving oxygen-mimetic radiosensitisers with radiotherapy (e.g., nimorazole); hypoxia-activated prodrugs (e.g., tirapazamine, evofosfamide); and small molecule inhibitors of hypoxia-relevant molecular targets (e.g. belzutifan, SLC-0111) (74). Overall, there is currently a clinical need to measure tumour hypoxia.

Currently, the most widely studied approach for assessing hypoxia in cancer patients involves endogenous markers, which has the advantage that large retrospective studies can be carried out (51, 54, 75). The approach uses immunohistochemistry on pre-treatment diagnostic biopsies (76). The markers used are hypoxia inducible *in vitro*, e.g., HIF-1α, CA9 and GLUT1 (76, 77). Many reports link high tumour HIF-1α expression with a poor prognosis including meta-analyses of studies in brain (n=1,422) (78), breast [BCa] (n=6,201) (79), digestive system (n=5,964) (80), hepatocellular [HCC] (n=3,570) (81), and oral cavity (n=1,471) (82) cancers. HIF-1α expression has been shown to be increased independently of hypoxia in clear cell renal carcinoma (ccRCC) due to a mutation in the von Hippel-Lindau (VHL) gene. The mutation inactivates normoxic proteasomal degradation of HIF-1α inducing elevated levels of HIF (83). Therefore, expression of hypoxia-related endogenous probes may not necessarily correlate with hypoxia and, are not a good standalone hypoxic marker. Meta-analyses have also shown high tumour expression of CA9 is an adverse prognostic factor in multiple cancers: renal cell carcinoma [RCC] (n=2,611) (84), oral squamous cell carcinoma [OSSC] (n=1,616) (85), and head and neck [H&N] cancer (n=1,470) (86). Several meta-analyses also show GLUT1 is an adverse prognostic factor in several cancers: lung (n=1,423 (87) and n=1,665 (88)), BCa (n=1,861) (89), colorectal cancer [CRC] (n=2,077) (90), and OSCC (n=1,301) (91). Furthermore, two meta-analyses in mixed cancer types showed high GLUT1 expression in tumours is associated with a poor prognosis (n=4,079 (92), and n=4,794 (93)). Although some of these markers have shown promise in hypoxia detection and stratifying patient therapy, this method relies on standardisation and establishment of guidelines across different laboratories, a common limitation in tissue-specific biomarkers (94). More focus is needed on systems that can be implemented into clinic (11).

Alternative measurements of hypoxia have also been developed including the indirect measurement of hypoxia using exogenous probes such as 2-nitroimidazole compounds (pimonidazole and EF5). These probes diffuse passively across the cell membrane and the nitro-group is enzymatically reduced to a reactive species inside the cell. Under hypoxic conditions, the nitro species undergoes further reduction and forms covalent bonds with central macromolecules which results in the accumulation of 2-nitroimidazole in hypoxic cells. However, within normoxic cells, the nitro species is re-oxidised and can diffuse out the cells (95, 96). Tumour biopsies are collected and hypoxia is detected using monoclonal antibodies. Multiple studies have shown the prognostic significance of these markers in H&N cancers, PCa, sarcomas, laryngeal cancer (LC), and glioblastoma (GBM). These markers are associated with locoregional control (LRC) (97), aggressive phenotype (98), metastases (99), poor outcome (100) and short time to recurrence (101).

Imaging-based markers for hypoxia are undergoing development such as ^18^F-MISO PET which uses a radioactive tracer, fluoromisonidazole (FMISO), to measure the levels of oxygen within tumours alongside positron emission tomography (PET) imaging. This is associated with survival outcomes and treatment response. In prospective hypothesis- generating and validation cohorts, patients with H&N cancer received ^18^F-MISO PET at different timepoints before or during chemoradiotherapy (CRT). Tumour hypoxia after 2 weeks of CRT correlated with a low LRC, whereas patients with oxic tumours had a good prognosis (102, 103). Carles et al. (104) prospectively analysed 35 HNSCC patients evaluated with ^18^F-MISO PET during CRT, correlating the changes in size and location of hypoxic areas within the tumour by a new classification parameter. The classification parameter distinguished between patients who had early or late disease progression, and how their hypoxic regions changed during CRT. Some of the radiomic features, particularly the low grey-level zone emphasis was able to predict local recurrence with high accuracy. Therefore, it was concluded that ^18^F-MISO PET hypoxia scanning has potential to be useful for personalised treatment plans and outcome prediction in HNSCC patients. However, ^18^F-MISO can bind to non-hypoxic cells in conditions of inflammation, infection or oxidative stress (105). Moreover, ^18^F-MISO has a slow blood clearance and tissue uptake therefore, it takes a long time to reach stable distribution in tumours (106). Although in a meta-analysis study, it has been shown that PET measured hypoxia is a robust parameter with a strong impact on outcome of HNSCC and that the most commonly investigated tracers ^18^F-MISO and FAZA (18F-Fluoroazomycin-arabinosid) can probably be used equivalently in multicentre trials (107). Dynamic contrast enhanced-MRI (DCE-MRI) has been shown to reflect heterogenous tumour perfusion and subtle tumour volume change during radiation/chemotherapy in prospective analysis of 62 CC patients. DCE-MRI is associated with tissue oxygenation and therefore, can be a good parameter for assessing hypoxia. In CC patients, they showed that there are independent and better predictors of tumour recurrence and death than clinical prognostic factors. Combining clinical prognostic factors and MRI parameters improves early prediction of treatment failure. This offers the potential of altering the treatment plan for patients (108).

Each of the techniques described have individual advantages and disadvantages (**Table 3**). The ability to measure hypoxia would aid clinical decision-making, with a robust predictive biomarker being the holy grail. However, a robust biomarker is not sufficient for its implementation into clinic, as shown by the oxygen electrode studies. Feasibility, economic costs, and undesirable effects in patients also need to be considered.

## Hypoxia and the tumour microenvironment

**Figure 1:**
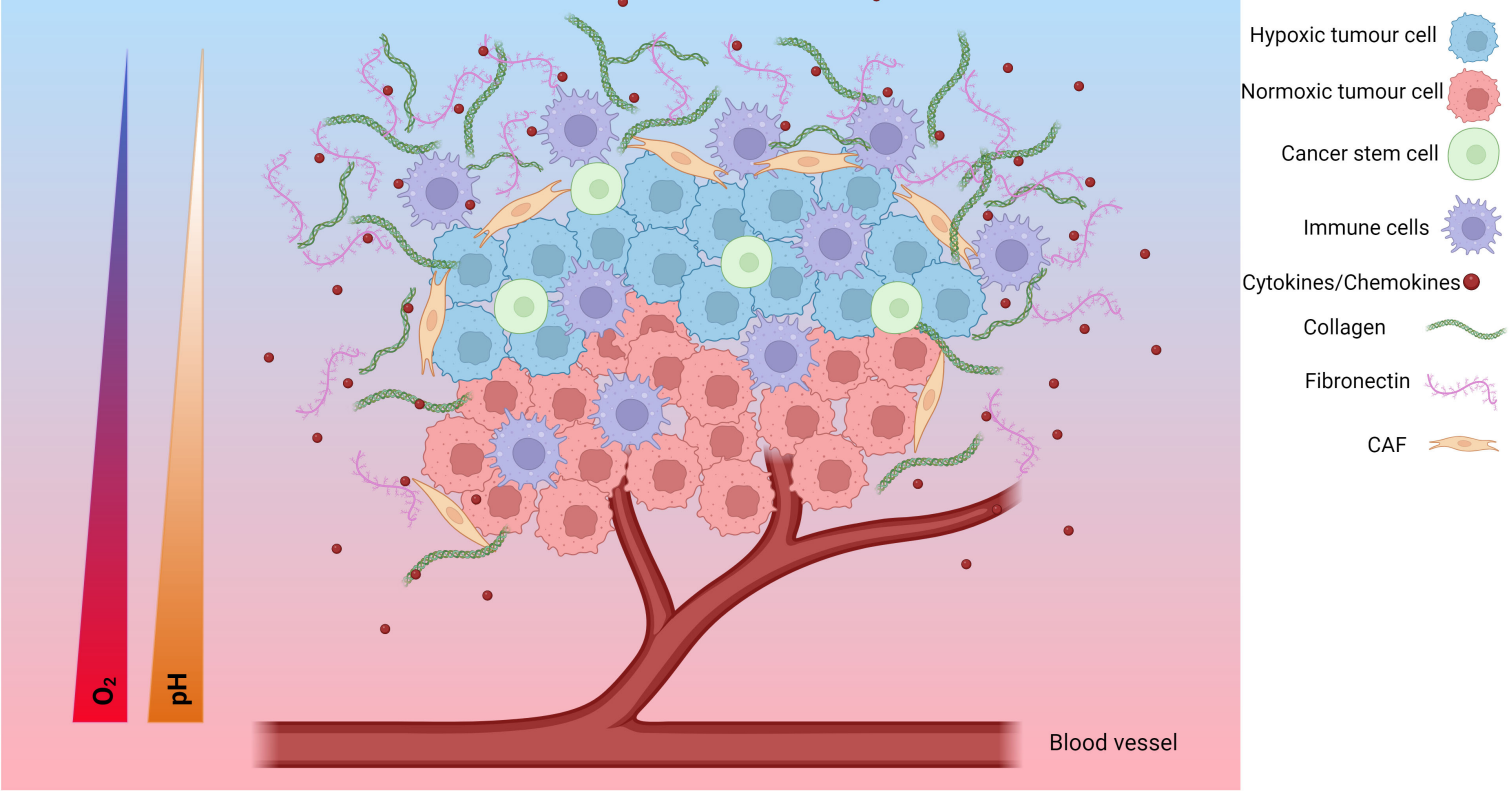
The hypoxic tumour microenvironment (TME). Hypoxia plays an important role in the development of the TME. The TME is composed of hypoxic tumour cells, cancer stem cells, tumour cells, immune cells, cytokines/chemokines, collagen, fibronectin, cancer associated fibroblasts (CAFs), endothelial cells and, blood vessels. As the tumour cells grow, the tumour cells further away from the blood supply have limited access to oxygen and become hypoxic tumour cells. Additionally, the TME undergoes a metabolic switch to meet the demands of the TME which involves increased glucose uptake and production of lactate resulting in an acidic TME characterised by a decreasing pH. These changes result in a change in cytokine/chemokine release, a change in immune cell phenotype, modification of the extracellular matrix, and activation of CAFs. Together, these form a more pro-tumorigenic environment prone to increased invasive and metastatic potential, as well as increased resistance to chemoradiotherapy. Created with BioRender.com.

The TME is a complex environment formed around tumours involving the interplay of several cell types and molecules. In solid tumours, the TME consists of cancer cells, surrounding blood vessels, immune cells, cancer-associated fibroblasts (CAFs), signalling molecules and the extracellular matrix (ECM) (**Figure 1**). The tumour type, stage of cancer and location can influence the nature of the TME. Tumour cells can change the composition of the microenvironment by releasing extracellular signals that promote angiogenesis or induce peripheral immune tolerance to evade immune detection (109). Features including hypoxia, the metabolic microenvironment, acidic niche and mechanical environment also play important roles in the phenotype of the TME (110). The hypoxic TME can help the tumour grow, spread or become resistant to treatments.

Metabolic switch within the TME is essential for cancer cell growth and metabolism, and adaptation to the hypoxic microenvironment. This phenomenon is described as the Warburg effect and is characterised by cell metabolism favouring glycolysis to meet the demand of the cancer cells for survival. The Warburg effect involves increased rate of glucose uptake and preferential production of lactate. This induces acidification of TME from accumulation of large amounts of metabolic waste products including lactic acid, carbon dioxide and bicarbonate protons (111). These products lower the pH of the extracellular space and create a gradient between the intracellular and extracellular pH. This has an impact on multiple hallmarks of cancer including suppression of immune response, increasing tumour invasion and metastasis, and modulating proliferation (112). Hypoxia also enhances upregulation of glucose transporters including GLUT1 and enzymes of the glycolytic pathway (e.g. CA9) feeding back into the glycolysis cycle (113, 114). A further consequence of acidification is the enhanced activity of matrix metalloproteinases (MMPs) which have a role in degrading the ECM and basement membrane (115). Therefore, the invasive and metastatic potential of tumours under hypoxia is enhanced. Acidification can also enhance resistance to chemotherapy and radiotherapy by reducing the uptake of drugs and activating DNA repair mechanisms (83).

This review will focus on the ECM, CAFs and immune cells within the hypoxic TME, both as individual components and how they interplay to create a more pro-tumorigenic microenvironment that can be manipulated for better patient outcome.

## Hypoxia and the extracellular matrix in cancer

The ECM is a complex network of biomolecules in the extracellular region that provides structural and mechanical support to surrounding cells. Several reports show its importance in the development of cancer (116). The ECM comprises a wide range of molecules that include structural proteins (e.g. fibronectin [FN], collagen [COL]), signalling molecules including cytokines and growth factors, (e.g. endothelial growth factor [EGF], transforming growth factor β [TGF-β]) and enzymes (e.g. MMPs, lysyl oxidase [LOX], prolyl 4-hydroxylase [P4HA]). The main protein components of the ECM are FN, COL, elastin and laminin proteins (117–121).

(116) CAFs are located in the tumour stroma and are the main producers of ECM (122–124). However, cancer cells also produce ECM and are important in determining the tumour ECM composition (125). In cancer development, the normal ECM phenotype shifts towards a cancerous ECM phenotype (126, 127). During this process, the ECM undergoes increased remodelling and deregulation of the levels of growth factors and enzymes (e.g. MMPs and TGF-β). Because of this remodelling process, a more fibrotic and stiffened ECM develops in cancer (116, 127). The transformation of the ECM towards a cancerous phenotype is a key process for tumour development that promotes cell growth and survival, metastasis and recruitment of cancer-associated cells (e.g. CAFs and tumour-associated macrophages [TAMs]), and modulates immune responses (4, 126, 128).

Hypoxia enhances ECM remodelling as a driver of tumour desmoplasia, a complex process that includes ECM degradation, composition and structural changes, generating a fibrotic and stiffer ECM (129). Degradation of the basement ECM membrane is enhanced in hypoxia as it drives neoplasia and tumour re-oxygenation (130). As reviewed by Chang and Chaudhuri (131), degradation of the basement membrane is a key for tumour progression that promotes local tissue invasion, and tumour metastasis (131). BCa patients with no basement membrane degradation have five-year overall survival (OS) rate of 99%. However, the percentage drops down to 85% for patients with local invasion of the basement membrane, and 27% once metastatic (132). Hypoxia increases MMPs secretion to induce basement ECM membrane degradation through HIF signalling (133, 134). For example, HIF-1 activity drives MMP2, MMP9, MMP14 and MMP15 overexpression (129, 134, 135). MMP2, MMP9, and MMP14 target and promote COLIV degradation, an activity associated with the destruction of basement membranes in BCa (136). MMP1 is also overexpressed due to HIF-1 activity in bladder cancer (BLCA), leading to increased migratory capacity in the presence of reactive oxygen species (ROS) (137).

The changes in the composition and structure of the ECM under hypoxia are driven by increased deposition and crosslinking of COL, FN and hyaluronic acid (HA) (138). Higher numbers and crosslinking of ECM fibres increase ECM stiffness and induces fibrosis. Fibrosis is associated with metastasis and poor cancer prognosis (139, 140). For example, fibrosis reduces the expected survival time of patients with non-small cell lung carcinoma (NSCLC) by 60% (141). Mechanistically, fibrosis promotes cancer development and spread through enhancing integrin mechanosensory pathways that activate EMT transition (e.g. FAK/Rho/ROCK signalling) (139, 140) leading to high ECM stiffness, fibrosis, and increased metastasis (129). Fibrosis induction is mostly mediated through increased COL deposition and enhanced expression of ECM remodelling enzymes (e.g. P4HA1, P4HA2, PLOD2) (129). Hypoxic induction of COL1 deposition has been known for 40 years and confirmed in several studies (142–145). Other studies also show HIF-mediated deposition and/or gene expression of fibrillar COLs (COL3 (146), COL5 (147–150), COL11 (149, 151) and COL27 (149)), basement membrane COLs (COL4 (150, 152, 153), COL7 (154), COL10 (151, 155) and COL18 (153, 156)), filament-forming COLs (COL6 (146)), fibril-associated COLs (COL9 (150, 151), COL14 (157)) and transmembrane COLs (COL13 (150)). Recent studies show hypoxia can also enhance fibrosis through increasing FN expression (158, 159). Increase in HA under hypoxia was first reported by Gao et al. (160). The findings were recently validated by Chen et al., who associated the increase in HA to higher invasive capacity of GBM cells (161). However, the role of HA in hypoxia-induced fibrosis has not been widely explored, and requires further research.

Enhanced deposition of ECM proteins (e.g. COL, FN, HA) is not sufficient to induce fibrosis. ECM crosslinking enzymes such as LOX are required to stiffen the ECM (129, 162). Increased expression of the LOX family members under hypoxia has been reported in several cancer studies (138, 163, 164), stromal cell, and endothelial cells (165). Similarly, hypoxia has been reported to promote the expression of other COL crosslinking enzymes through HIF signalling (PLOD1 (166), PLOD2 (167), P4HA1 (149, 168–170) and P4HA2 (149, 169)).

**Figure 2:**
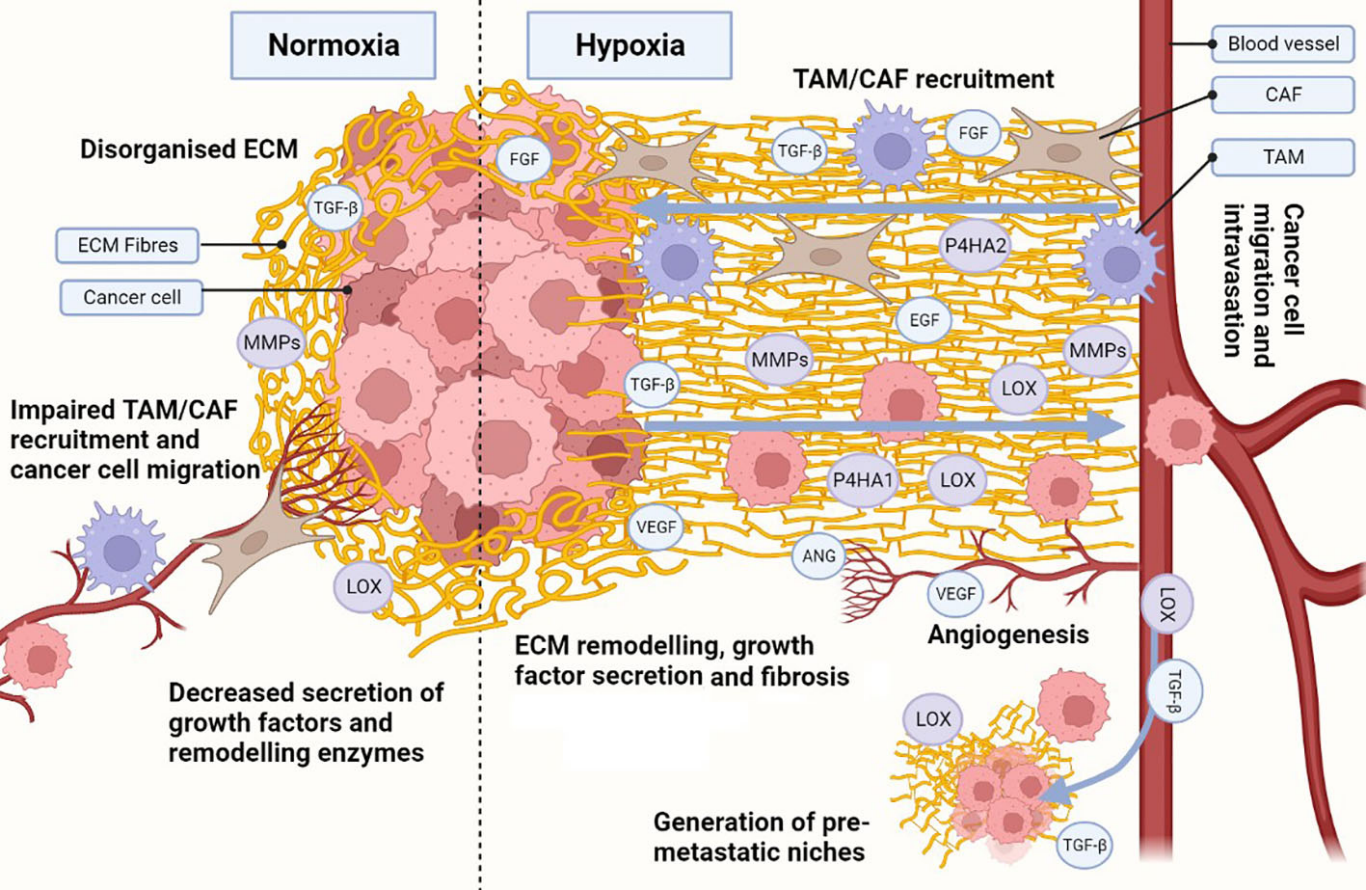
Hypoxia influences the development of a cancerous ECM. Hypoxia in the ECM increases collagen (COL) and fibronectin (FN) deposition, as well as secretion of metalloproteinases (MMPs), lysyl oxidases (LOX) and prolyl 4-hydroxylase subunit alpha (P4HA) 1 and 2. Increased MMPs, LOX and P4HA1/2 promote the generation of organised aligned COL and FN fibre tracks in the ECM, enhancing cell migration. Hypoxia also induces the secretion of growth factors and cytokines, which are also released due to ECM remodelling, establishing a synergistic effect. Release of growth factors (e.g. transforming growth factor β [TGF-β], endothelial growth factor [EGF], fibroblast growth factor [FGF]) enhance not only cancer cell growth and survival, but also recruitment of cancer-associated fibroblasts (CAFs) and tumour associated macrophages (TAMs). TAMs and CAFs participate in the secretion of growth factors, ECM remodelling and COL/FN deposition, increasing the synergistic effect. Under hypoxic stress, angiogenesis is activated through secretion of angiogenic growth factors (e.g. vascular endothelial growth factor [VEGF], angiopoietin [ANG]). The angiogenic process allows for the development of new blood vessels, enhancing ECM remodelling during the process. Additionally, an organised hypoxic ECM provides migratory tracks directing cells towards blood vessels and enhancing intravasation. Pro-tumorigenic growth factors and enzymes (e.g. TGF-β, LOX) can intravasate and travel to distant healthy tissues, generating pre-metastatic niches through ECM remodelling. The same migratory tracks enhance cancerous cell migration and intravasation, allowing them to circulate and eventually colonise the pre-metastatic niches and seed new tumour cells. Created with BioRender.com.

We can infer that higher expression of crosslinking enzymes under hypoxia enhances COL and FN fibrogenesis, leading to a fibrotic and desmoplastic ECM in cancer, but it is currently believed that hypoxic fibrosis generates migratory tracks to promote cancer cell migration and metastasis (**Figure 2**). This model is supported by reports showing that the expression of ECM-crosslinking enzymes is necessary for metastasis in *in vivo* models, and is associated with worse patient outcome (170, 171). Hypoxia also increases the expression of integrin receptors, further enhancing the mechanosensory pathways driving EMT transition. Hongo et al., have shown hypoxia enhances cancer cell migration through upregulation of α2, α5 and β1 integrins (172). Ju et al., showed upregulation of integrins α1, α5, α11 and β1, with α5 and β1 integrin also increased cancer cell migration (173). As the integrins described above are COL and FN receptors, it is possible that their upregulation under hypoxia provides a migratory advantage in a context where hypoxia is inducing fibrosis due to increased COL and FN deposition and crosslinking. However, there is evidence in the literature contradicting the current model. Kakkad et al., and Goggins et al., have shown hypoxia reduces COL fibre density through HIF-1 signalling using *in vivo* BCa and PCa xenograft models (174, 175). Furthermore, Kuchnio et al., demonstrated that low prolyl-hydroxylase 2 (PHD2), a protein that induces HIF-1 hydroxylation and degradation, impairs ECM deposition, fibrogenesis, and metastasis (176). These findings were validated by Madsen et al., who showed PHD2 inactivation impairs CAF activation, ECM deposition, and fibrosis (177).

There are several possible explanations to the contradictory findings. Most studies addressing hypoxia in the ECM have been in fibroblasts, as they are considered the main drivers of fibrosis in cancer (139, 140). However, Tian et al., have shown cancer cells also change the ECM composition and remodelling in the development of fibrosis (178). Furthermore, the same study highlights fibroblasts and cancer cells have distinct contributions to the ECM during the development of fibrosis (178). Therefore, it is possible hypoxia drives distinct ECM remodelling process in fibroblasts and cancer cells.

Regarding the hypoxia mechanism driving ECM remodelling, most studies have focused on the role of HIF-1/2 (110). However, the role of HIF-3, the unfolded protein response (UPR) and the DNA damage response (DDR) pathways in the induction of fibrosis is poorly understood. Distinct levels of oxygen deprivation (179), and the length of exposure to hypoxia (3, 180) can change the signalling of the hypoxia pathways. Therefore, variability across experimental settings can also explain the differences reported in literature. In addition, to the current data no study has mechanistically proven hypoxias ability to enhance ECM fibrogenesis. Therefore, further clarification of the mechanisms driving hypoxia ECM remodelling, and its links with fibrosis and metastasis in hypoxic tumours is needed.

## Hypoxia and cancer associated fibroblasts

**Figure 3:**
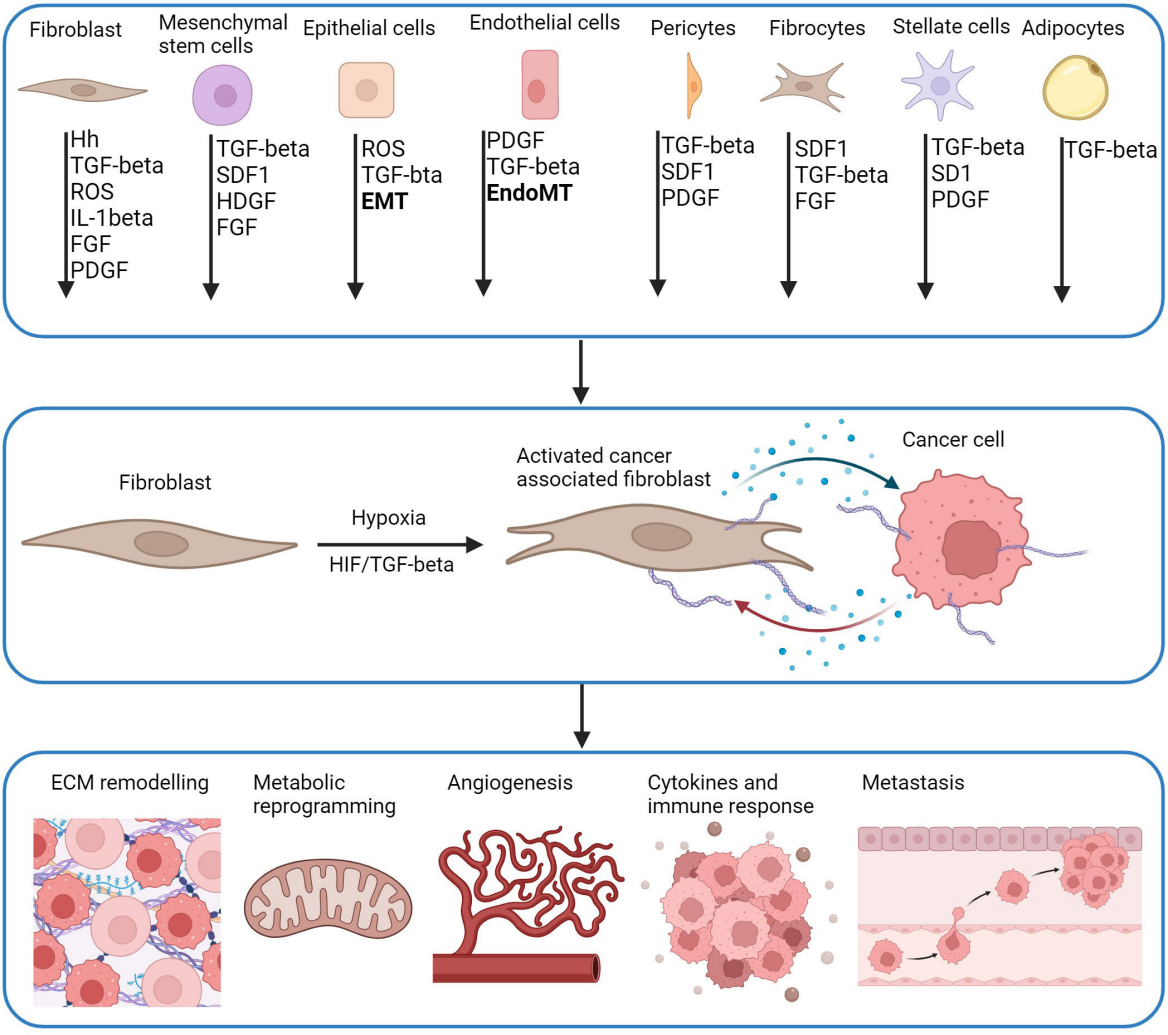
CAF activation pathways and downstream effects. CAFs are derived from multiple cell types and activated by multiple molecules including hedgehog (Hh), transforming growth factor-β (TGF-β), reactive oxygen species (ROS), interleukin-1β (IL-1β), fibroblast growth factor (FGF), platelet-derived growth factor (PDGR), stromal cell-derived factor 1 (SDF-1), heparin binding growth factor (HBGF) which can be driven by hypoxia. Precursor cells including mesenchymal stem cells (MSCs) are a source of CAFs activated by CXCL-12 and TGF-β derived from tumour cells. Pericytes, fibrocytes, stellate cells and adipocytes are also recruited by tumours by CXCL-12 and TGF-β, and are activated by TGF-β and PDGF. CAFs can derive from mature epithelial cells that differentiate into functional CAFs by TGF-β mediated epithelial-mesenchymal transition (EMT). Endothelial cells undergo EndoMT to differentiate into CAFs through TGF-β and SMAD signalling. HIF and TGF-β have a role in the function of CAFS. Genes associated with changes in ECM remodelling, metabolic reprogramming, angiogenesis, immune response and metastasis are direct transcriptional targets of HIF in CAFs or cancer cells. This creates bi-directional communication between CAFs and cancer cells through release of cytokines and chemokines (blue dots) which promotes proliferation of both cells, and further enhancement of pro-tumorigenic pathways (Adapted from (181, 182)). Created with BioRender.com.

CAFs are a type of stromal cell within the TME characterised by an elongated morphology. They can be derived from different cell types including resident fibroblasts, mesenchymal stem cells (MSCs), pericytes, smooth muscle cells, endothelial cells, epithelial cells, fibrocytes, stellate cells and adipocytes (**Figure 3**). CAFs are characterised by a lack of protein expression for epithelial, endothelial, or hematopoietic cells however, do express mesenchymal biomarkers including vimentin, α-smooth muscle actin (α-SMA), fibroblast activation protein (FAP) and platelet-derived growth factor receptor-alpha (PDGFR-α) (183). They can be regulated by both HIF-dependent and HIF-independent mechanisms (110).

CAFs interact with multiple TME cells and have a significant impact on tumour biology including angiogenesis, invasion, immune evasion, metastasis and drug resistance (183) (**Figure 3**). Hypoxia can alter the phenotype and function of CAFs, modulating the crosstalk between different cells of the TME (110). Due to their diverse role within the TME, tumour-promoting CAFs have been considered potential therapeutic targets in cancer (110, 183, 184). More recently, different CAF subtypes have been identified with either pro-tumorigenic or anti-tumorigenic effects (185). Although research is emerging within this field, the focus on hypoxia is limited. Therefore, it is important to differentiate between subtypes and cancer types, to deliver a more targeted and personalised therapy for patients.

### CAF activation by hypoxia

Fibroblasts are activated in response to multiple signalling molecules including TGF-β, interleukin-1β (IL-1β), PDGF, ROS, stromal cell-derived factor 1 (SDF1), sonic hedgehog protein (SHH), hepatoma derived growth factor (HDGF) and FGF. These can be secreted by both cancer cells and stromal cells (**Figure 2**). CAF activation can mediate cancer progression under hypoxic conditions (**Figure 3**), HIF/TGF-β activation regulate ECM remodelling, immune response, metabolic reprogramming, angiogenesis and metastasis (181).

Hypoxia activates resident fibroblasts specifically by ROS and the activation of the HIF-1α pathway which are both driven by hypoxia. Resident fibroblasts are stimulated and mobilised by TGF-β1 to activate CAFs (**Figure 3**). PDGF, FGF, SHH and IL-1β secreted by tumours, also play a role in the activation of resident fibroblasts into CAFs activating ERK, Shh/Smo and NFκB pathways. Pericytes, fibrocytes, stellate cells and adipocyte-derived CAFs are recruited by tumours by TGF-β and SDF-1, and are activated by TGF-β or PDGF. CAFs also originate from precursor cells that are recruited by tumour cells. These are activated by the multiple signalling molecules described. MSC-derived CAFs are activated by SDF-1,TGF-β, HDGF and FGF. Epithelial cells can differentiate into CAFs through TGF-β and ROS mediated EMT (181). Endothelial cells undergo a similar process known as EndoMT which is driven by TGF-β and PDGF (**Figure 3**) (186).

CAFs respond much like cancer cells to hypoxia and undergo metabolic reprogramming to adapt to the TME and to support glycolysis. CAFs also provide essential metabolites for tumour growth. They have a bi-directional role with cancer cells, and enhance tumour proliferation. The metabolic switch is important in transforming fibroblasts to CAFs (23). Both melanoma tumour cells and CRC CAFs undergo glycolysis under hypoxic conditions however, CAF proliferation decreases relative to primary foreskin fibroblasts. It is proposed that the increase in glycolysis is to aid proliferation of tumour cells (23). Becker et al. (187) show that hypoxia induces epigenetic reprogramming of normal fibroblasts which results in a pro-glycolytic CAF-like phenotype. This induces the metabolism of BCa cells and promotes tumour growth. Together, these data highlight the role of CAFs in promoting tumour growth, and how this can be driven by HIFs.

### CAF subtypes

Hypoxia induces oncogenic signals that can change both CAF phenotype and function reviewed by Han et al. (188). Rhim et al. (189) and Özdemir et al. (190) highlight that the lack of clinical success in targeting CAFs could be attributed to poor understanding of their heterogeneity. Therefore, further elucidation of this could help develop more targeted therapies.

These studies demonstrated that there is no common consensus for defining CAFs within literature. Without a standardised naming convention, it will be difficult to stratify patients who may benefit from CAF-targeting therapeutics. CAFs have a significant role in regulating multiple pro-tumorigenic pathways by the hypoxic TME (**Figure 3**). Although, it is important to fully understand the CAF/cancer cell axis, a deeper insight into CAF subtypes is needed to fully elucidate these pathways.

### CAFs and hypoxia

Contrary to data often presented on the role of HIF-1α as a tumour-promoting factor, Kim et al. (191) have shown that targeted deletion of HIF-1α in stromal cells enhances tumour growth. In mammary stromal cells, HIF-1α is a negative regulator of tumour development. HIF-1α null and VEGF-A-null mammary tumours were associated with reduced hypoxia and decreased permeability and density of tumours (191). TAM infiltration was reduced in both null mammary tumours. In contrast, Chiavarina et al. (192), showed that fibroblasts expressing HIF-1α increased xenograft MDA-MB-231 breast tumour volume by ~3-fold. This was associated with a reduction in caveolin-1 expression and an increase in aerobic glycolysis shown by loss of mitochondrial activity and increased lactate production. Direct activation of HIF-1α in MDA-MB-231 cells in a xenograft model, showed a 3-fold reduction in tumour volume. These data suggest that HIF-1α effects are cell type dependent. The fibroblasts analysed by Kim et al. (191), were derived from a mouse mammary model, compared to Chiavarna et al. (192) who used a human immortalised cell line, hTERT-BJ1.

In androgen-deprived prostate tumours, hypoxia is common and associated with activation of HIF-1 and induction of TGF-β expression. This results in differentiation of fibroblasts to myofibroblasts and production of CXCL13 which drives B-cell recruitment and a more aggressive phenotype. This was highlighted in a transgenic adenocarcinoma mouse model, where the emergence of castration-resistant PCa was inhibited by inhibition of TGF-β or blocking phosphodiesterase-5 which prevents activation of myofibroblasts and CXCL13 (193). These studies used different animal models, cancer models and methods which could cause discrepancies within the data. The genetic homogeneity within these species means that there is lack of genetic variation that is more representative of the human population. Most murine cancers are derived from mesenchymal origin whilst human tumours mainly arise from epithelial tumours (194). Additionally, the origin, differentiation and malignancy can change the nature of the interaction between HIF-1α and CAFs.

Despite the evidence that CAFs have a tumour-promoting role, there are studies that highlight tumour-supressing properties. It is unknown whether fibroblast subtypes are normal fibroblasts that are resistant to differentiating into CAFs, or a subtype of anti-tumour CAFs. Hu et al. (195) emphasise the challenge of personalising treatments due to our lack of understanding CAFs functional distinctions within patient tumours. However, they were able to identify 3 CAF subtypes from patients with NSCLC, the different subtypes correlated with clinical response to targeted therapies and tumour immune microenvironment. They showed that NSCLC cells with mutant *EGFR* which accounts for 20% of NSCLC resistance to tyrosine kinase inhibitors (TKIs) had differing responses to EGFR TKI, Osimertinib, dependent on CAF subtype.

Meflin is a protein expressed in CAFs within the pancreatic TME. Low Meflin expression correlated with straight collagen fibre alignment and a more aggressive pancreatic cancer (PaCa) phenotype (196). Lida et al., showed that Meflin positive CAFs have a tumour suppressive role in a PDAC mouse model. Am80 was identified as a reagent that induces Meflin expression in CAFs and increased PDAC sensitivity to chemotherapeutics. Furthermore, tumour vessel area and intra-tumoral drug delivery was enhanced following treatment with Am80. Meflin suppressed tissue stiffening by interacting with LOX to inhibit its COL crosslinking activity (197). CAFs are largely associated with creating an immunosuppressive TME, reducing the efficacy of immune checkpoint blockade. However, CAFs expressing Meflin in patients with NSCLC, showed enhanced survival and favourable therapeutic response to immune checkpoint blockade. Higher prevalence of Meflin-positive CAFs is positively correlated with CD4-positive T-cell infiltration and vascularisation within NSCLC tumour. The anti-tumorigenic role of Meflin positive CAFs has also been shown in CRC models (198). Takahashi et al., propose that hypoxia depletes expression of Meflin on CAFs resulting in cancer-promoting CAFs that induce chemoresistance (199).

Brechbuhl et al., show that estrogen-receptor (ER+) BCa have two CAF subtypes defined by their CD146 expression. CD146^neg^ CAFs supress expression of ER and are less sensitive to tamoxifen. Whilst CD146^pos^ CAFs maintain ER expression in BCa cells and sustain estrogen-dependent proliferation and sensitivity to tamoxifen. This is also shown in PaCa (200), blocking CD146 in CAFs significantly enhanced tumour cell migration and invasion in co-cultures with PaCa cells (201).

There has been limited research on the role of hypoxia in regulating CAF phenotype. Madsen et al., show that chronic hypoxia and HIF-1α stabilisation deactivate CAFs in an orthotopic BCa model. The loss of PHD2 activity following chronic hypoxia prevents both CAF-induced ECM remodelling and BCa metastasis (177). This suggests that hypoxia may have a positive interaction with CAFs, and that the influence of hypoxia differs between different cell types. Despite the evidence of both pro-tumorigenic and anti-tumorigenic roles of CAFs, there is limited research on the role of hypoxia in regulating CAF expression and role within the TME. All these data imply that targeting HIF-1α and its signalling pathways in CAFs may provide different outcomes between different patients and cancers. Therefore, it is essential that specific characteristics of individual patient tumours and CAFs is taken into consideration rather than a one-size-fits-all approach.

## Hypoxia and immune cell regulation

Several infiltrated immune cells can be found within the TME including CD8 T cells, CD4 T cells, regulatory T cells (Tregs), natural killer cells (NK), TAMs and dendritic cells (DCs) (202). Under physiological conditions, a tightly regulated balance between immune activation and immune suppression is needed to maintain homeostasis (203). External factors including hypoxia can alter this balance (204). Data supports the role of hypoxia in dampening the anti-tumour immune response by modulating key processes including immune cell activation, infiltration and function (205). Hypoxia-induced immune modulation can occur via direct and indirect mechanisms including changes in cytokines and growth factors, expression of immune checkpoint molecules and metabolic activity (206–209). The role of hypoxia in modulating immune cell activity is complex and may be tumour-dependent or person-dependent. In addition, hypoxia can have different effects on the distinct immune cells present within the TME.

### Regulation of T cells

T cells (CD8+ and CD4+) are an important component of the adaptive immune system and play a key role in eliminating tumour cells via various cytotoxic activities (210, 211). The anti-tumour function of T cells can be suppressed in several ways. This includes up-regulation of inhibitory checkpoint molecules and the presence of other immune cells such as Tregs, TAMs and myeloid-derived suppressor cells (MDSCs) (210). Evidence suggests that hypoxia plays an important role in inducing T cell dysfunction by modulating expression of checkpoint molecules and immune cell infiltrate via changes in growth factors, cytokines, chemokines and intra-tumoural pH (212–214).

The presence of hypoxia in tumours causes up-regulation of inhibitory immune checkpoint molecules and growth factors that reduce the cytotoxic and survival potential of T cells (212–222). Hypoxia has been shown to up-regulate immune checkpoint molecules on T cells (TIGIT, TIM3 and VISTA) in pre-clinical models of melanoma, CRC and lung cancer. They are associated with reduced anti-tumour T cell activity (6, 159, 213, 215–222).

VEGF is a hypoxia-responsive growth factor that has a role in immune regulation. *In vivo* studies investigating the effects of inhibiting VEGF on immune regulation suggest that blocking VEGF improves the cytotoxic potential of CD8+ T cells by increasing production of IFNγ and TGF-β (223, 224). However, careful consideration of the anti-VEGF approach needs to be highlighted as although inhibiting VEGF improved anti-tumour immune cell phenotype, this was accompanied by increased hypoxia. Using combinational therapies may help eliminate the risk of negative side effects. In addition, targeting hypoxia via VEGF or HIF-1α may need a deeper understanding of the cell-type or tumour expression profile. Especially since Palazon et al. (224) show that deletion of either HIF-1α or VEGF in CD8+ T cells reduces their ability to infiltrate and kill BCa tumours.

Single-cell analysis of tumours from HCC patients demonstrates that regions of high hypoxia have increased Treg infiltration and decreased granzyme B positive T cells compared with low hypoxic regions (214). *In vitro* analysis suggested that hypoxia increases secretion of CCL28 which increases Treg migration (212). In addition, the hypoxic TME is more acidic and glucose deprived which leads to changes in immune cell metabolism (i.e. activation of glycolysis, increased lactic acid levels and amino acid metabolism) (24, 207–209, 225–231). These metabolic changes provide Tregs with a survival advantage, as they are resistant to high levels of lactate. Meanwhile, other immune cells including NK cells, DCs, CD8+ and CD4+ T cells cytotoxicity and maturation is inhibited, causing increased immune suppression (207–209, 226, 231–233). *In vitro* co-culture analysis using HCC cell lines suggests that hypoxia increases expression of indoleamine 2, 3-dioxygenase 1 (IDO1) in monocyte-derived macrophages and Tregs leading to reduced CD8+ T cell proliferation and cytotoxic effects and expansion of Tregs (227, 229). The effects of hypoxia on IDO1 are potentially cell-type specific as expression of IDO1 in tumour cells (i.e. OC cells) is reduced under hypoxic conditions (229). This along with the differences observed in targeting VEGF and HIF-1α in different cell types highlights the complexity associated with drug-targeting. Suggesting that a greater understanding of the wide-spread expression profile of targets of interests is needed to improve drug efficacy and reduce toxicity.

### Regulation of NK cells

NK cells form part of the innate immune response that can induce rapid and strong anti-tumour activity without the need for priming by antigen-presenting cells (211). However, under hypoxic conditions expression of PD-L1 on NK cells is increased, leading to reduced CD8+ T cell proliferation (234, 235). In addition, hypoxia reduces NK cell activation in response to tumour cells by reducing the expression of NK cell surface receptors (i.e. NKp46, NKp30, NKp44 and NKG2D) (236). Although hypoxia reduces expression of receptors associated with NK cell activation, the surface density and function of the Fc-γ receptor CD16 remains unaffected under hypoxia (236), suggesting that hypoxia does not affect the cytotoxic potential of NK cells. Similar to observations in T cells, hypoxia affects NK cell activity to different extents and may be tumour-dependent, person-dependent or dependent on the severity of hypoxia (236–239). Balsomo et al. (236) found that even in the presence of hypoxia, NK cells were able to efficiently kill melanoma cells but the same was not observed for HCC and BCa cell lines (237, 238). *In vitro* analysis of HCC cell lines demonstrated that HCC cells and NK cells exposed to 6%, oxygen significantly inhibited NK cell toxicity against the HCC tumour cells (238). However, at oxygen concentrations <6% there was no significant decline in NK cell activity, suggesting differences in downstream pathways activated at different oxygen concentrations. This is an important observation as it suggests that the extent of hypoxia can influence the resulting NK cell response either positively or negatively. Therefore, biomarkers that allow for gradients of tumour hypoxia to be identified would be advantageous in providing personalised treatment approach to targeting immune cells.

### Regulation of myeloid-derived suppressor cells

MDSCs are immune cells derived from the myeloid linage that have potent immunosuppressive activity even under physiological conditions (240). Hypoxia increases intra-tumoural lactate, promoting production of MDSCs which reduces T cell and NK cell proliferation and cytotoxic potential of T cells. Noman et al. (241) showed that hypoxia upregulates the expression of PD-L1 but not PD-L2, PD-1, CTLA-4, CD80 or CD86 on splenic MDSCs. Subsequent inhibition of PD-L1 increased the cytotoxic capability of CD4+ and CD8+ T cells by restoring the production of IFNγ. Down-regulation of IL6 and IL10 is the proposed mechanism for improved T cell cytotoxicity by PD-L1 blockage (241).

### Regulation of monocyte-derived tumour-associated macrophages

TAMs are innate immune cells that are recruited into the tumour and differentiate from monocytes. Recruitment and differentiation of TAMs is orchestrated by chemotactic signals from soluble factors such as CCL2, CCL5, colony-stimulating factor 1 (CSF1), VEGF, semaphorin 3A (SEMA3A) endothelial cell monocyte-activating polypeptide-II (EMAP-II), endothelin, stromal cell-derived factor 1α (SDF1α) and oncosatin M (211, 242). Within the tumour, TAMs are found in abundance in both vascular and avascular stromal areas. TAMs can exist as M1 or M2 phenotype, with M2 TAMs associated with a pro-tumorigenic potential. Hypoxia modulates TAM recruitment, phenotype and function to induce an immunosuppressive TME.

Hypoxia inhibits the mobility of TAMs, resulting in accumulation of TAMs within hypoxic areas. Sica et al. (243) showed that defective expression of CCR2, a monocyte chemotactic protein in TAMs derived from patients with OC induces monocyte migration towards CCR2. Chemokines and cytokines produced by TAMs are key modulators of angiogenesis and metastasis, promoting a pro-tumorigenic microenvironment. TNF-α and IL-1 are secreted by macrophages and blood monocytes (244, 245). Both cytokines are associated with stimulating VEGF, and therefore may drive angiogenesis (246, 247).

Increased lactic acid in regions of hypoxia induces an M2 TAM phenotype which is associated with poor prognosis in BCa, CC, PCa and BLCA (231, 248–250). Exosomes secreted from hypoxic tumours contain elevated levels of cytokines and chemokines that drive macrophage recruitment and M2 polarisation. Park et al. (251) showed that exosomal protein secretion in hypoxic melanoma, squamous skin carcinoma and lung cancer cells was 3-4 fold higher than in normoxic cells. The hypoxic exosomes were associated with secretion of immunosuppressive mediators including TGF-β1and chemo-attractants including, CSF-1 associated with M2-like macrophage polarisation. The role of exosomes in promoting M2 polarisation has been demonstrated further by Shou et al. (252) in oesophageal cancer. Downregulation of PTEN is alsoshown by Zhu et al. (253) via exosomal miR223 derived from hypoxic TAMs co-cultured with OC cells. This resulted in decreased apoptosis, increased cell viability and enhanced drug resistance. In cancer, an M2-phenotype macrophage can enhance immunosuppression, promote angiogenesis and drug resistance. Polarisation of TAMs to an M2-like phenotype induces secretion of IL-10, TGF-β or VEGF which inhibit T cell function, induce Tregs and promote angiogenesis.

iNOS expression is increased in the presence of HIF-1α in macrophages under hypoxic conditions (24). Doedens et al. (24) show that hypoxia mediates suppression of T cell infiltration *in vitro* dependent on macrophage expression of HIF-1α in BCa models. IDO expression has been shown to increase monocyte-derived macrophages in a CCL20 dependent manner when co-cultured with hypoxic HCC cells. This correlated with increased HIF-1α expression. Additionally, the monocyte-derived macrophages were shown to supress T cell function and induce Tregs, generating an immunosuppressive microenvironment (227).

### Regulation of monocyte-derived dendritic cells

DCs are specialised antigen presenting cells that differentiate from circulating monocytes and link the adaptive and innate immune response (211). Low oxygen tension has been associated with the differentiation and function of DCs.

Hypoxia reduces the ability of DCs to uptake tumour antigen and downregulates the expression of DC differentiation and activation markers including CD40, CD80 and MHCII via increased production of factors including IL-10, iNOS and VEGF. This affects the ability of DCs to process and present tumour antigens, reducing T cell priming and ultimately the induction of treatment induced immunogenic cell death (254). In addition, hypoxia induces increased secretion of osteopontin by DCs, a factor associated with enhanced migration of tumour cells (255).

### Regulation of immune cells by hypoxia via other cells in the TME

There is strong evidence for the role of hypoxia in inducing direct effects on immune cells. However, hypoxia can also modulate immune cells indirectly via actions of tumour cells, endothelial cells and CAFs.

In terms of immune regulation by tumour cells, hypoxia has been shown to modulate epithelial mesenchymal plasticity. In lung adenocarcinoma, hypoxia-induced a mesenchymal phenotype in some hypoxic tumour cells. These hypoxic subclones demonstrated increased resistance to CD8+ T cell and NK cell-mediated lysis via TGF-β signalling (256). *In vitro* data using human cell lines derived from metastatic PCa and BCa tumours (DU145 and MDA-MB-231) demonstrates that hypoxia (0.5% O_2_) increases tumour cell expression of PD-L1 in a HIF-1α dependent manner leading to increased T cell apoptosis (257). *In vivo* models of melanoma suggest that increased expression of NANOG under hypoxic conditions upregulates the expression and secretion of TGF-β, and promotes the infiltration of immunosuppressive cells (258). *In vivo* models of BCa suggest that hypoxic mammary tumours secrete a variety of cytokines and growth factors (CCL2, G-CSF, TNF-α, VEGF, TIMP-1 and MMP-9) that increase infiltration of MDSCs (CD11b^+^/Ly6C^med^/Ly6G^+^). This increase in myeloid cell infiltration is inversely correlated with NK cell suppression, which enabled a microenvironment primed for metastatic growth of disseminated tumour cells within the lung. HIF-1α expression has been shown to negatively correlate with major histocompatibility complex (MHC) class I chain-associated genes which are essential for tumour antigen presentation and immune recognition. *In vitro*, analysis of PaCa demonstrated that hypoxia reduces the expression of MHC on the surface of PaCa cells. In addition, hypoxia induced the shedding of membrane bound MHC into extracellular space forming a soluble MHC which acts as a decoy and reduces the capacity of antigen presenting immune cells to present antigens to CD8+ T cells, thereby reducing T cells priming and T-cell mediated killing.

Hypoxia can induce changes to endothelial cells via increased expression of VEGF and FGF, altering interstitial pressure, intra-tumoural perfusion and expression of adhesion molecules. Changes in adhesion molecules (i.e. ICAM1, P-selectin, E-selectin, MAdCAM-1 and VCAM) on endothelial cells can lead to reduced immune cell infiltration and shift the balance of the infiltrated immune cell profile towards a more immune suppressive phenotype.

Hypoxia also modulates chemokine and cytokine production by CAFs, which increases immune cells associated with an immunosuppressive microenvironment. Data demonstrates that hypoxia increases CAF secretion of CXCL13 which promotes B cell recruitment in PCa. Increased B cell tumour infiltration is associated with progression to castration-resistant disease and neuroendocrine differentiation, both of which are associated with poor prognosis (193). In BCa, hypoxia-induced CAF secretion of CXCL12 suppresses the anti-tumour activity of T cells, DCs, NK cells and enhances the pro-tumour activity of Tregs, MDSCs and TAMs. Hypoxia up-regulates TGF-β secretion which up-regulates expression of PD-L1 and PD-L2 on CAFs. This promotes T cell exhaustion and immune evasion (259).

## The tumour microenvironment and resistance to therapy

The ECM, CAFs and immune cells are all shown to be impacted by hypoxia, having an effect on cancer cell resistance to chemotherapy, radiotherapy and immunotherapy. One way in which hypoxia induces resistance is by modulating the TME. This phenomenon has attracted research interest for a long time, yet it still remains unsolved (260, 261).

Our understanding to date includes knowledge of the hypoxic TME inducing cell quiescence and causing resistance of tumours to cell-cycle specific drugs including alkylating agents (e.g cisplatin), antimetabolites (e.g gemcitabine), mitotic inhibitors (e.g paclitaxel) and cyclin-dependent kinase (CDK) inhibitors (e.g Palbociclib) (262–266). Hypoxia can also activate survival pathways including PI3K/AKT, MAPK and NFκB which induces resistance to apoptosis and DNA damage summarised by Rohwer and Cramer (267). Hypoxia is also a well reported factor contributing to poor response to radiotherapy (1, 268, 269). and suppression of immune response by activation of expression of immune checkpoint inhibitors. It also alters the composition and function of immune cells within the TME (213, 270).

### Resistance to chemotherapy

Resistance to chemotherapy is controlled by multiple mechanisms, and these effects are often enhanced by hypoxia. Biomechanical and biophysical properties of the ECM of solid tumours often induce resistance to chemotherapy. Hydroxylation of collagen by P4HA1, P4HA2, PLOD1 and PLOD-2 mediates tissue stiffness and is regulated by HIF-1 (110). Hayashi et al. (271) highlight that increased tissue stiffness is associated with a poorer clinical complete response in BCa patients, relative to patients with a low tissue stiffness score (10% versus 38% respectively). This has also been shown in PaCa by Rice et al. (272), where matrix stiffness was correlated with chemoresistance to paclitaxel. Matrix stiffness has been proposed to enhance chemoresistance by inducing cell cycle arrest in the G_0_ phase. Physical signals induced by matrix stiffness are transduced to tumour cells via integrin receptors which have a role in mechano-transduction. This alters cell morphology, proliferative capacity and invasive ability of tumour cells. However, there is conflicting data on the exact role of matrix stiffness in therapeutic efficacy dependent on the tumour type. In metastatic CRC, increased ECM stiffness was regulated by highly activated metastases-associated fibroblasts which reduced the efficacy of bevacizumab. Addition of anti-RAS, a hypertension drug, to the regime enhanced response to bevacizumab by reducing stiffness of the ECM (273). Qin et al., highlight how intermediate matrix stiffness (38 kPa) induces resistance to doxorubicin measured by cell death rates (29.6% cell death) in BCa cells (MDA-MB-231), compared to low matrix stiffness (10 kPa- 48.5% cell death) and high matrix stiffness (57 kPa- 55.2% cell death). It is proposed that high expression of integrin-linked kinase (ILK) and translational coactivator, Yes-associated protein (YAP), within the BCa matrix is associated with resistance to doxorubicin (274). In contrast, MCF-7 BCa cells exposed to a rigid matrix stiffness (2710 kPa), the IC50 of cisplatin and taxol decreased significantly (p < 0.01), compared to the soft matrix stiffness (5.3 kPa) which showed more resistance. OC cells, SKOV3, were also shown to have enhanced survival within the soft matrix (0.5 kPa), compared to a stiffer substrate (25 kPa) following treatment with 1 μM cisplatin. This correlated with overexpression of multi-drug resistance proteins, ABCB1 and ABCB4, in cells grown on the soft gel matrix (275). Furthermore, in osteosarcoma cell lines, the IC50 value and viability of cells was significantly higher at 7kPa compared to the 55 kPa matrix (276). Bordeleau et al., have correlated increased matrix stiffness with disruption of vessel architecture and integrity and promotion of tumour-like vascular phenotype. Increased stiffness of collagen resulted in increased outgrowth of angiogenic sprouts from spheroids, and a 1.5-fold increase in branching, compared to the softer collagen gels. Furthermore, they demonstrated that MMPs played a key role in promoting increased angiogenesis which was found in stiffer matrices. This provides a potential explanation for reduced chemotherapeutic efficacy following increased matrix stiffness (277). Overall, these data demonstrate the contradictions in data, and that it is important to consider multiple components of the matrix including stiffness and its effects on vasculature. These data have all been demonstrated in a normoxic context however, we know hypoxia has an essential role in regulating matrix stiffness (110), and therefore is likely to modulate response to chemotherapy.

Another consequence of a hypoxic TME is its acidification, this is detrimental to resistance to therapies through a mechanism described as ‘ion trapping’ (278). The phenomenon of ‘ion trapping’ is described as weak bases isolated in acidic compartments, and weak acids sequestering into alkaline compartments. This has consequences for weak base drugs including anthracyclines and vinca alkaloids (279). Furthermore, Wachsberger et al. (280) showed that chronic exposure to acidic conditions activates heat-shock protein, HSP-27, inducing cisplatin resistance. Vukovic & Tannock (281)., observed that intracellular acidification arrested cells in G_1_ phase, making the cells more resistant to mitoxantrone, paclitaxel and topotecan in murine mammary carcinoma cells and urothelial cancer cell lines. These chemotherapeutics are all weak bases therefore, acidic conditions reduce the cytotoxicity of these drugs by inhibiting their uptake due to a larger proportion of the drug molecules becoming protonated, limiting diffusion to cells.

The cell cycle plays an important role in regulating cancer cell proliferation and apoptosis. The hypoxic TME induces cell cycle arrest or quiescence in cancer cells (260). This is particularly prevalent in the G_1_/S and G_2_/M checkpoints, which protect cells from DNA damage and genomic instability. Cells become “stuck” in these cell cycle phases making them less sensitive to chemotherapies that target rapidly dividing cells. Chronic hypoxia is associated with the induction of a quiescent state in cancer cells, where the cells are temporarily and reversibly arrested in G_0_ phase. This is associated with a more aggressive tumour phenotype (282). The G_0_ phase of the cell cycle is where cells are not actively dividing. These cells remain dormant until the cells are exposed to favourable conditions (283). Druker et al., reviewed the role of hypoxia and its control of the cell cycle (284) highlighting that identifying G_0_-arrested cells within tumours remains a challenge as there is a lack of easily measurable markers to measure this state (283). BCa cells exposed to chronic hypoxia (1% O_2_ for up to 7 days) were shown to enter G_0_/G_1_ cell cycle phase (282), and it is established that this phase of the cell cycle is resistant to cytotoxic chemotherapies (285).

p16^INK4A^ has been related to inducing cell senescence, and inhibition of pRb phosphorylation through cyclinD/CDK4 (286). Box et al. (287) measured cell cycle arrest genes in multiple cancer cell lines and primary fibroblasts. They found that different cell types had different cell cycle arrest profiles in response to hypoxia. BCa cells (HTB-30), CC cells (HeLa) and human mammary epithelial cells all showed induction of G_1_/S arrest after initial exposure to hypoxia. However, a HCC cell line (Hep3B) lacked observable G_1_/S arrest in hypoxia conditions. Although all cell lines showed reduced proliferation at 24 h. Loss of multiple cyclin-dependent kinase inhibitors (CDKI) was found in the cell lines including p16, p21 and p27. Methylation of *P16* has been associated with reduced sensitivity to paclitaxel in patients with advanced NSCLC (288). Therefore, regulation of CDKI by hypoxia plays a role in resistance to chemotherapeutics. Yano et al. (285) highlighted that 90% of cancer cells within the centre of tumours are in G_0_/G_1_ phase following implantation of MKN45 metastatic stomach adenocarcinoma cells in nude mice. Furthermore, 75% of cancer cells located >100 µm from tumour blood vessels are also in this phase. Therefore, most drugs currently used in clinics are ineffective in solid tumours as they target cancer cells in S/G_2_/M phases.

Dysregulation of DNA repair pathways is associated with initiation and progression of cancers. Hypoxia increases DDR proteins, decreasing homologous directed repair by downregulating BRCA1, BRCA2 and RAD51, decreasing mismatch repair proteins MLH1, MSH2, MSH3 (289) and downregulating base excision repair factors including, APE1, OGG1 and MYH (290). With these mechanisms active within hypoxic tumours treated with chemotherapies, it can be difficult to effectively treat solid tumours. PARP inhibitors have shown promise in overcoming dysregulation of DNA repair pathways (291), Shelton et al. (292) showed *in vitro* and *in vivo* enhancement of the effects of 5-FU, irinotecan or oxaliplatin and radiation with PARP inhibitor, ABT-888 in CRC cells. However, hypoxia has been associated with reduced efficacy of PARP inhibitors in multiple cancer cell lines (293). Dysregulation of DNA repair pathways can have significant implications for patients, which leads to genomic instabilities and accumulation of mutations. Understanding DNA repair deficiencies in tumours can aid in treatment decisions including the use of PARP inhibitors. Other targets such as DNA-dependent protein kinase inhibitors and Rad51 inhibitors are currently being explored preclinically. Prognostic biomarkers can indicate whether a patient is suitable for these treatments by considering mutations in BRCA1/BRCA2 in BCa which indicates a more aggressive phenotype. Identifying these mutations can inform clinicians that patients will be suitable for treatment with PARP inhibitors (294). Weil et al., show that dependent on the DNA repair pathway defects, breast and ovarian tumours can be more sensitive with platinum-based drugs or PARP inhibitors (295). This demonstrates that identifying patients with DNA repair defects can guide treatment decisions. In BRCA deficient cells, PARP inhibition is 3 times more effective than cisplatin. Lou et al., highlight the benefits of profiling patients with DDR pathway profiling. Patients with a low DDR score did not benefit from adjuvant chemotherapy with anti-PD1 (296).

Many chemotherapies induce apoptosis of cells however, resistance to chemotherapy is characterised by a reduction in apoptosis. The B-cell lymphoma-2 protein (Bcl-2) family of proteins are one of the main regulators of the intrinsic apoptosis pathway, and in chemoresistance upregulation of Bcl-2 proteins is observed which offsets the pro-apoptotic proteins. Hypoxia has been indicated as a key regulator Bcl-2 proteins, downregulating pro-apoptotic proteins and upregulating anti-apoptotic proteins in HCC and lung cancer cell lines (297). Treatment of OC cell lines with cisplatin was associated with upregulation of Bcl-x_L_ and subsequent chemoresistance (298). Additionally, dysregulation of inhibitors of apoptosis (IAPs) have been correlated with chemoresistance in multiple cancers. IAP overexpression of cIAP1 in oesophageal squamous carcinoma correlated with resistance to cisplatin and camptothecin (299). Overexpression of ML-IAP (Livin) correlated with resistance to etoposide, vincristine, 5-FU in CRC cells (300). In primary cells derived from melanoma patients, Nachmias et al. (301) also showed resistance to etoposide correlating with increased expression of the gene. These data were associated with patient clinical response.

Our knowledge on the dysregulation of apoptosis in solid tumours has led to the exploration of novel drug compounds that increase apoptosis (**Table 4**). There are multiple molecules within the apoptotic pathway that can be targeted; agonists to TRAIL and SMAC mimetics have been developed. Birinapant has been tested in patient-derived xenograft models of OC and CRC and melanoma showing growth inhibition following intraperitoneal administration (30mg/kg) (302). Tolinapant (ASTX660) is another SMAC mimetic and IAP antagonist which has shown success preclinically in HNSCC, BLCA and CRC (303). In H&N cancer, tolinapant enhanced radiation-induced immunogenic cell death in syngeneic mouse models. An *in vitro* BLCA study showed that tolinapant induced necroptosis. It was proposed that this mechanism could help overcome resistance to cisplatin in BLCA (303). Crawford et al. (304) show that there may be clinical benefit of combining tolinapant with FOLFOX chemotherapy in microsatellite stable CRC with elevated cIAP1 and cIAP2.

**Table 4:** Clinical trials targeting different apoptotic pathways within solid tumours.

Compound	Molecular target	Clinical trial phase	Disease sites targeted	Main findings	Refs
ONC201	DRD2- TRAIL induction	I	Refractory solid tumours	Well tolerated and biologically active in advanced cancer patients	(58)
ONC201	DRD2- TRAIL induction	II	Recurrent/Refractory metastatic breast cancer and advanced endometrial carcinoma	ONC201 was tolerable but did not have significant clinical activity as a monotherapy	(59)
ONC201	DRD2- TRAIL induction	II	Neuroendocrine tumours	Tolerated well in patients with metastatic neuroendocrine tumours. Showed clinical benefit*	(60)
ONC201	DRD2- TRAIL induction	II	Recurrent glioblastoma	Median OS was 41.6 weeks. One patient had durable OS with 85% regression in one lesion and 76% regression in the second lesion. Another patients continues to receive ONC201 for >12 months and remains disease-free	(61)
Eftozanermin alfa	TRAIL-receptor	I	Advanced solid tumours	Acceptable safety, evidence of pharmacodynamic effects and preliminary anticancer activity.	(62)
LCL161	IAPs	I	Solid tumours	Well tolerated up to doses of 1800 mg	(63)
LCL161	IAPs	I	Relapsed/refractory small cell lung cancer and gynaecologic cancer	Study stopped before the maximum tolerated doses and recommended phase II dose. Addition of oral topotecan causes more myelosuppression and did not improve outcome	(64)
LCL161	IAPs	II	Triple negative breast cancer	TNFα gene signature was predictive of sensitivity of patients to LCL161 in combination with paclitaxel	(65)
Birinapant	IAPs	I	Solid tumours	Maximum tolerated dose (47 mg/m^2^), safety and pharmacokinetic properties confirmed. Prolonged stable disease in 3 patients, and accumulates in tumour cells results in downregulation of cIAP1.	(66)
Birinapant	IAPs	II	Relapsed/refractory metastatic colorectal cancer	Birinapant + irinotecan showed clinical benefit with the greatest benefit in KRAS mutated colorectal cancer	(67)
ASTX660	IAPs	Phase I/II	Advanced solid tumours	Data published on lymphoma showing a manageable safety profile and clinical activity at 180-mg/day. Data not published on solid tumours	(68)
Xevinapant	IAPs	Phase II	Advanced squamous cell carcinoma of the head and neck	Combination with CRT demonstrated superior efficacy. Probability of survival 5 years after randomisation was 53% in patients treated with xevinapant + CRT vs 28% in placebo CRT arm	(69)
APG-1387	IAP	I	Advanced solid tumours	Well tolerable with manageable adverse events	(70)
SurVaxM	Survivin	I	Recurrant malignant glioma	Well tolerated. 3 patients maintained partial clinical response or stable disease for + 6 months. Median progression free survival was 17.6 weeks. Median OS was 86.6 weeks.	(71)
Navitoclax (ABT-263)	Bcl-2 inhibitor	I	Small cell lung carcinoma	Safe and well tolerated with dose dependent thrombocytopenia as the major adverse effect. 1 x patient had a partial response longer than 2 years. 8 patients had stable disease.	(72)

*Clinical benefit was considered maintenance of performance status with no new metastases in 3 months.

Myeloid cell leukemia-1 (Mcl-1) is a member of the anti-apoptotic Bcl-2 family, and has been identified as an apoptotic survival factor in TNBC. Mcl-1 is commonly amplified in 56% of TNBC tumours and its overexpression associated with poor clinical prognosis (305). This was one the studies that led to the investigation of Mcl-1 as a target for patients with poor prognosis. Pre-clinical models in PaCa, BCa, lung cancer and OC cell lines showed Mcl-1 inhibition *in vivo* and *in vitro* (306–308). Both preclinical studies and clinical trials show that modulation of apoptosis is a valuable target in tumours with dysregulated apoptosis, and offers a strategy for overcoming chemoresistance.

Hypoxia upregulates expression of drug efflux pumps and confers with resistance to chemotherapy. Multidrug resistance protein 1 (MRP1), multidrug resistance-associated protein 1 (MRAP1) and breast cancer resistance protein (BRCP) are all regulated by HIF-1α in CRC (309) and HIF-2α in OC (310). Additionally, reduced drug uptake due to poor blood supply is characteristic of hypoxic tumours (311). There are limited studies that correlate clinical data with chemotherapy response. One of the proposed reasons for limited knowledge on MDR1/P-gp expression is poor sensitivity and specificity and difficulty in quantifying levels of protein by immunohistochemistry, and normal tissue contamination (312). Furthermore, clinical trials targeting drug efflux pumps show little promise of improving patient outcome. Despite attempts to target efflux pumps that are upregulated in cancer cell lines, there is limited efficacy in the drugs developed. Mohelnikova-Duchonova et al. (313) show that in PaCa, multiple ABC transporters are upregulated compared to normal tissue. This highlights that tumours are likely to adapt to alternative mechanisms of resistance if single efflux pumps are targeted (313). In contrast, A phase I trial combining tyrosine kinase inhibitor, pazopanib, with topotecan which is an approved treatment for SCLC, CC and metastatic OC showed a 1.7-fold increase in patient exposure compared to topotecan treatment alone. Pazopanib was trialled due to its mild affinity for Pgp/ABCB1 and high affinity for BCRP/ABCG2 (314).

### Resistance to radiotherapy

Approximately 50% of all cancer patients undergo radiotherapy as part of their treatment, 60% with curative intent (315). Radiotherapy uses high-energy radiation to target and destroy cancer cells. There are two main ways of delivering radiotherapy to patients; external beam radiation which uses an external source of radiation to target tumours, or internal radiation therapy including brachytherapy which uses radioactive sources including seeds, wires or pellets that are placed directly inside or near the tumour (316). However, it was discovered by Gray et al. (317) that tumour cells are less damaged by a dose of X- or γ-radiation than oxygenated cells versus anoxia at the time of irradiation.

Other forms of radiotherapy include protons, electrons and carbon ions. Proton beam therapy delivers a higher dose of radiation than conventional radiation using photons whilst sparing the surrounding tissue. However, it is only used in a subset of patients, particularly children and patients with complex brain cancer, H&N cancers and sarcomas in the UK. Treatment is limited to these tumour types as proton beam therapy requires a high degree of accuracy and precision which means that tumours that change shape or move due to close proximity to organs of the autonomic system, are unsuitable for this therapy. It is beneficial to use proton beam therapy in children as its localised effects reduce late-stage toxicity (318). Dose painting can be used to target more hypoxic regions of tumours with a higher dose of protons, to try and overcome the potential of radio-resistance and induce permanent damage to DNA of cancer cells (319).

Oxygen is an essential factor that influences response to radiotherapy in solid tumours. Gray et al. (317) showed that when partial pressure of oxygen is below 20mmHg at the time of irradiation, the cells become resistant to radiation damage. When tumours are targeted with radiation, they absorb the radiation and produce highly reactive free radicals either directly or indirectly. The radicals produced are unstable and highly reactive with oxygen, which induces damage to the target tissue. Radiation induces DNA damage by producing ROS and inducing apoptosis. Therefore, presence of oxygen in tumours enhances the effect of radiation by increasing ROS production and inhibiting DNA repair. To produce the same effect in hypoxic conditions, 2.5-3.0-fold increase in radiation is required (320). Horsman et al. (75) review and summarise the clinical impact of hypoxia in patient outcome following radiotherapy (321–323). As previously described, solid tumours have varying degrees of tissue oxygenation that fluctuates overtime. Therefore, when radiotherapy is delivered during periods of reduced oxygen, its effectiveness is reduced (324). Thiruthaneeswaran et al. (325) summarise the role of hypoxia on radiotherapy and the challenges that remain within the field that need to be overcome to offer patients the best clinical outcome.

Understanding the hypoxic TME is crucial for optimizing treatment outcomes. Hypoxia alters radiation effectiveness, necessitating higher doses for hypoxic tumours, yet this can lead to increased side effects (326). Approaches including altered fractionation, dose escalation, and high linear energy transfer (high-LET) radiation help combat hypoxia’s challenges. To improve efficacy, radio-sensitizing agents like nitroimidazoles or carbogen can be employed. Wang et al. stress the need for precise dose guidelines in clinical practice. Overall, tailoring treatment plans to individual tumour biology remains key for achieving optimal outcomes (327).

When discussing radiotherapy, we often refer to the 6R’s of radiotherapy which are of mechanisms that are important in determining the response of biological tissue to multiple doses of radiation. These include repair, redistribution, repopulation, reoxygenation, radiosensitivity and reactivation of the immune system (328–330). Hypoxia plays a role in dysregulating the 6R’s and impacting patient response to treatment.

Disruption of the 6 R’s by hypoxia has been discussed by Rakotomalala et al. (50). Wozny et al. (331) show that hypoxia (1% O_2_) increases non-homologous end-joining (NHEJ) and increases radio-resistance in HNSCC. Hypoxia disrupts the capacity of cells to repair radiation-induced DNA making hypoxic cells less susceptible to damage by radiation. Primary human fibroblasts exposed to chronic hypoxia (0.2% O_2_) were shown to have defective repair of DNA double-strand breaks (DSBs) following irradiation, compared to cells grown in normoxic conditions. It was proposed that unrepaired DNA-DSBs drive genetic rearrangement and genomic instability under hypoxic conditions. Genomic instability can increase metastatic capacity of tumours. The authors propose that understanding the DNA-DSB repair defects regulated by hypoxia in tumours could improve the treatment modalities used for cancer (332). Exposing H1299 lung carcinoma cells to chronic hypoxia (72 h 0.2% O_2_) downregulated homologous recombination (HR) proteins which increased sensitivity to DNA cross-linking agents and increased radiosensitivity compared to acutely hypoxic cells (6h 0.2% O_2_) and anoxic cells. Therefore, chronically hypoxic cells that are repair deficient may be a novel target to selectively kill hypoxic cells (333). BCa cells cultured in 3% O_2_, showed reduced expression of *RAD50*, *RAD51*, *BRCA1* and *BRCA2* which are essential for HRR. The same genes were downregulated in PCa with the addition of *RAD54*, and NHEJ genes (*Ku70, LIG4* and *XRCC4*) when cultured in 0.2% O_2_ (334, 335).

Redistribution describes alterations to the cell cycle following radiotherapy. Hypoxic cells enter arrest in G_1_/S and G_2_/M phases making cells less sensitive to radiation, hindering the redistribution of cells to more sensitive cell cycle phases. Enhanced expression of HIF-1α correlates with upregulation of p21 and CDKI-1 phosphorylation in PCa. Luo et al. (336) showed that this correlates with radioresistance both *in vitro* and *in vivo* which maintains PCa cells in G_0_/G_1_ or S phase.

Repopulation following radiotherapy describes the proliferation of cancer cells that have survived irradiation. Hypoxic conditions often drive rapid proliferation and therefore the accelerated growth reduces the efficacy of radiotherapy. Cancer stem cells play an important role in recurrence of tumours following radiotherapy. Luo et al. (336) showed that WNT/β-catenin signalling is responsible for progression of PCa tumours following radiotherapy which is driven by HIF-1α and is responsible for repopulation. In gastric cancer, hypoxia has been shown to increase KDM4B (lysine demethylase) which induces cyclin A1 expression following irradiation. This enhances cancer cell proliferation following treatment (337).

**Figure 4:**
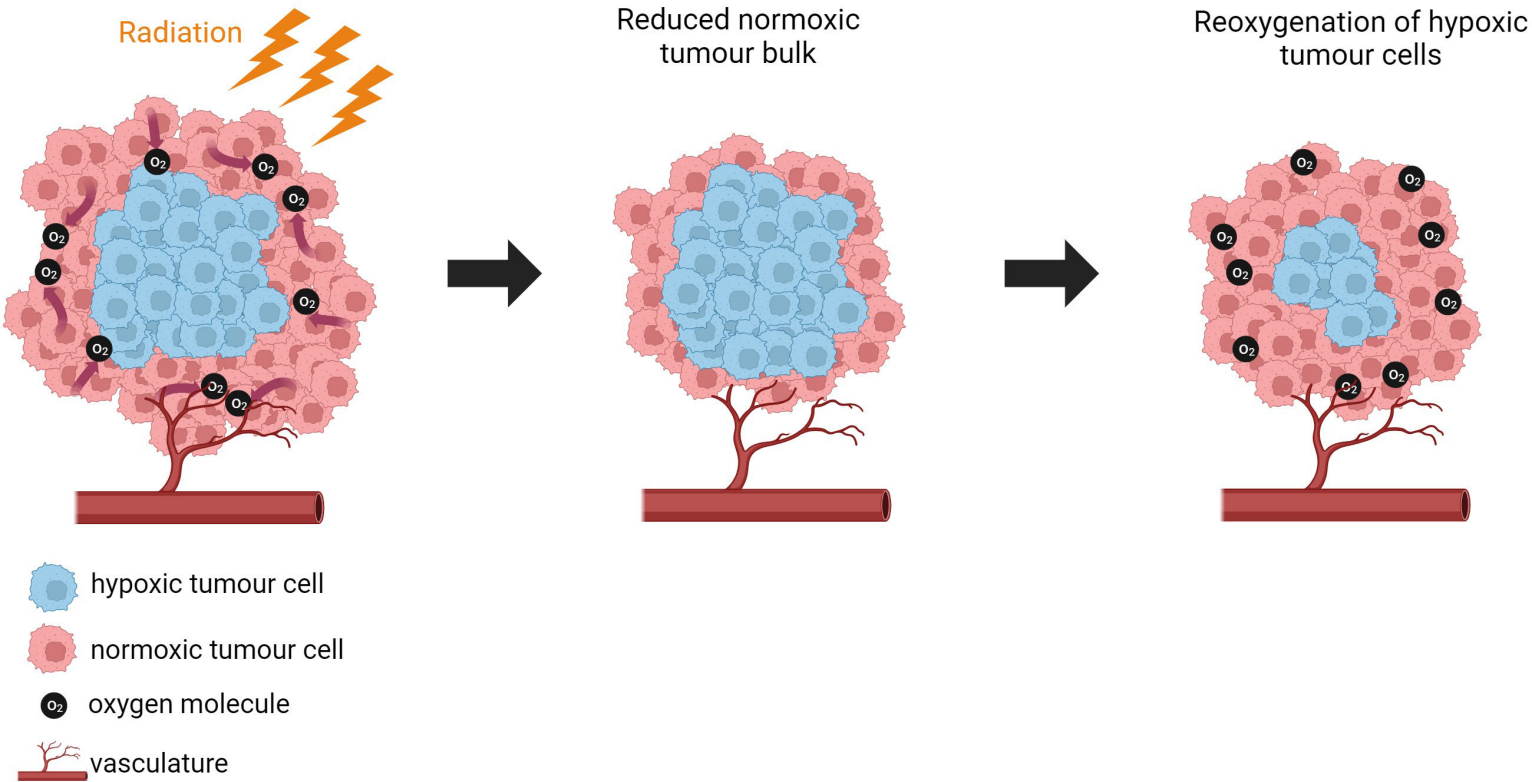
Tumour reoxygenation. Normoxic tumour cells are killed by irradiation which induces a reduction in tumour bulk. Oxygen consumption is reduced by normoxic cells which enables oxygen to diffuse to hypoxic regions. This induces vessel regrowth and reoxygenation of hypoxic cells. The reoxygenated cells are more sensitive for reirradiation. Created with BioRender.com.

Reoxygenation of tumour cells occurs between radiotherapy fractions. Reoxygenation describes when death of oxygenated cells within normoxic regions decreases the oxygen consumption within these areas which enables molecular oxygen to diffuse to hypoxic regions that are located 70-100 micrometres from functional blood vessels. More hypoxic tumours with disrupted vasculature struggle to reoxygenate and therefore make the cells more resistant to radiotherapy (**Figure 4**). Adding treatments to a patients plan which enhances oxygen delivery can help overcome this mechanism. Moeller et al. (338) show that through a poorly understood mechanism, tumours exposed to radiation release cytokines that inhibit apoptosis of endothelial cells. This is regulated by HIF-1, and enhances oxygen delivery to tumour cells that survive initial irradiation. In a CC model, tumour cells rapidly repopulated following irradiation as the decreased tumour bulk caused by the initial dose, reduced tumour mass and created a favourable growth environment. This was mediated by Akt/mTOR dependent mechanisms, which activated HIF-1 intra-tumoural activity (339). HIF-1 stabilisation in these tumours further enhances radioresistance. Increased lactate levels induced by metabolic switch in HNSCC and CC are associated with reduced sensitivity to radiotherapy (340, 341). Autophagy has been attributed to a cytoprotective role, protecting cells from damage including radiation. This has been shown in osteosarcoma, BCa and rectal cancers (342, 343).

The mechanisms of apoptosis activated during cell death can impede cancer cell radiosensitivity. Hypoxia directly modulates the cancer cell apoptotic response, which reduces cancer cells radiosensitivity. Cuisnier et al. (344) showed that chronic hypoxia (3% or 5% O_2_) led to overexpression of Bcl-2 in CC cells (KB-3-1). Overexpression of Bcl-2 inhibited radiation induced apoptosis by inhibiting ROS production. Furthermore, expression of the pro-apoptotic molecule, Bax, was reduced with no translocation of the gene in the mitochondria. Decreased Bax expression mediated by HIF-1 was also shown by Bamodu et al. (345) in HCC which correlated with a radiation-resistant phenotype. This mechanism driven by PDK1 which is a HIF-1α target gene which drives PI3K/AKT/mTOR pathway, inducing radiotherapy resistance. Rakotomalala et al. (50) summarise further mechanisms of resistance to cell death.

Hypoxia and immune cell regulation play a role in the anti-tumour microenvironment therefore, the ‘6^th^ R’ was identified by Boustani et al. (328). It describes the reactivation of the anti-tumour immune response following irradiation. Radiotherapy induced tumour cell death releases tumour-associated antigens, activates DCs and produces cytokines and chemokines that stimulate the priming and infiltration of T cells into the tumour. However, within a hypoxic TME, the immunogenicity of tumour cells reduces by increasing immunosuppressive cells. There are currently ongoing trials combing radiotherapy with immunotherapy (328). More recently, a ‘7^th^ R of Radiobiology’ has been described by Taghizadeh-Hesery (346) as ‘reinforcement’ by the TME. This describes that radiation cancer cell response can be altered by both cellular and noncellular components that surround the tumour, and this can be regulated by hypoxia (346).

### Resistance to immunotherapy

As researchers have gained more insight into the crucial role of anti-tumour immunity and hypoxia in patient prognosis, immunotherapies have been introduced as a therapeutic intervention for some solid tumours (270, 347). HIF-1α upregulates PD-L1 on both tumour and stromal cells, whilst its receptors PD-1, CTLA-4 and LAG3 are all expressed on immune cells. Overexpression of PD-L1 is associated with resistance to immunotherapies in hypoxic melanoma, CRC and glioma. Combining metformin, which reduces oxygen consumption with anti-PD-1 therapy in melanoma and CRC showed improved efficacy in hypoxic tumours. Inhibiting HIF-1α in glioma reduced PD-L1 expression and enhanced immunotherapy efficacy (348, 349). Furthermore, retrospective analysis of HNSCC patient samples treated with immunotherapy either as front-line therapy or following platinum failure found that patients, with higher %CA9/mean intensity (%CA9/I) associated with more hypoxic tumours, had reduced efficacy of anti-PD-1 therapy. Metastatic/recurrent patients with a lower % CA9 were associated with improved OS, low CA9/I was associated with a 12-month OS rate of 51.3% versus 14.1% in patients with high CA9/I. Patients with low hypoxia and high CD8 had better efficacy with anti-PD-1 (350). Upregulation of HIF-1α/CXCL12 correlates with higher PD-L1 expression in HCC, and therefore worse prognosis in these patients (351).

## Pseudohypoxia and cancer

Oxygen-dependent HIF activation has been extensively covered in this review, but it is important to consider oxygen-independent HIF activation leading to pseudohypoxia. Interest is growing in this concept as it may explain the persistence of the Warburg effect in cancers where even non-hypoxic cells preferentially use glycolysis for ATP production (271). A number of gene mutations have been associated with this phenomenon. These gene mutations have led to the development of compounds for clinical trials and approval of antagonists for multiple solid tumours, as one of the implications of pseudohypoxia is resistance to conventional therapies (352, 353).

### Von Hippel-Lindau

Mutations in the VHL gene result in the loss of the Von Hippel-Lindau tumour suppressor protein (pVHL), which is responsible for targeting HIF for degradation. Without functional pVHL, HIF remains stabilized, contributing to the pseudohypoxic state and the development of various tumours (354). In the case of ccRCC, this results in a highly angiogenic tumour due to overproduction of hypoxia inducible VEGFA mRNA. Initial VHL inactivation in RCC induces expression and accumulation of both HIF-1α and HIF-2α however, HIF-2α expression becomes dominantly expressed in chronic hypoxia and supresses HIF-1α protein (355). This promotes oncogenic potential by driving tumour progression and metastasis through activation of hypoxia-sensitive signalling pathways and overexpression of HIF-2α target genes (352). The understanding of the biology underpinning this phenomenon has produced early positive results using a HIF-2α antagonist to treat ccRCC, resulting in approval by the U.S. Food and Drug Administration (FDA) (353).

### Mouse double minute 2 homolog (MDM2)

Data from Retinoblastoma and Myelodysplastic syndromes (MDS) implicate MDM2 in pseudohypoxia. Zhang et al., have shown that MDM2 promotes cell survival in retinoblastoma through regulating both pVHL and HIF-1α resulting in HIF stabilisation (356). Studies in MDS suggest that TP53 loss of function mutations can affect oxygen-independent HIF degradation by MDM2. This results in accumulation of HIF-1α and pseudohypoxia (357). These data suggest that novel agents such as MDM2 inhibitors could have a role in the treatment of cancers with a number of clinical trials under way (358).

### Other genetic factors

Pseudohypoxia can also be driven by genetic mutations or alterations in other genes involved in the oxygen-sensing and response pathways (359). Mutations in genes associated with angiogenesis such HIF-dependent neovascularisation through VEGF or through anti-apoptotic proteins such as IAP-2 can result in activation of hypoxia-associated pathways (360). Understanding the molecular basis of oxygen-independent pseudohypoxia will lead to novel target and biomarker discovery and potential for improved patient outcomes.

## Clinical trials

This review has defined the biological importance of tumour hypoxia with its ubiquitous effects. The clinical importance of hypoxic regions within solid tumors has been known since the early 20th century. Data from numerous studies reveal the prevalence of hypoxia in various types of human tumours, though there is significant variability among individual cases.

Over the past four decades, controlled clinical trials have demonstrated that radiation resistance due to tumour hypoxia can be mitigated by interventions like normobaric or hyperbaric oxygen therapy and the use of nitroimidazoles as hypoxic radiation sensitizers. More recently, hypoxic cytotoxins, drugs that selectively target cells in hypoxic environments, have gained attention.

As far back as 2008, a systematic review involving 10,108 patients across 86 randomized trials aimed to modify tumour hypoxia in patients receiving primary radiation therapy found that hypoxic modification significantly improved the efficacy of radiotherapy (361). Hypoxic modification resulted in better LRC and an associated improvement in OS. However, the incidence of distant metastases and radiation-related complications did not show significant changes. There are only two solid tumours in which hypoxia modification is standard of care. HNSCC where the ARCON Trial and DAHANCA 5 trial confirmed that hypoxia modification improved LRC (97, 323). In the ARCON trial, LRC improved by 80% in patients with T3 and T4 laryngeal cancer which aided in organ preservation (97). Within the DAHANCA-5 trial, LRC increased to 49% with addition of nimarazole versus 33% in the placebo group (323). Since DAHANCA-5 randomised patients between radiotherapy alone and radiotherapy with nimorazole, nimorazole is used as a standard of care in Scandanavia. The BCON trial confirmed that radiotherapy with carbogen and nicotinamide was superior to radiotherapy alone for muscle-invasive BLCA with a 13% absolute improvement in OS (362). Updated 10-year outcomes show that benefit is maintained especially if patients are stratified by hypoxia biomarkers such as necrosis or a 24-gene transcriptomic signature (363).

More recently, Bourigault et al., have shown that the antimalarial drug atovaquone can reduce tumour hypoxia detected using hypoxia PET in NSCLC patients. This is a small promising phase II study which poses the challenge of how investigators pursue clinical trials without industrial support (364).

Pseudohypoxia may prove to be an area of greater interest with industry already pursuing clinical trials with a novel molecular agent targeting HIF-2α approved by the FDA based on data from a small phase II study while the phase III trial is awaited (353).

Despite the substantial evidence supporting the benefits of hypoxic modification, its implementation in clinical practice remains limited. There are many reasons why this is including prejudice, lack of familiarity and funding structures (365).

## Conclusions and future perspectives

Hypoxia is a key regulator of the TME and plays a role in regulating hallmarks of cancer (366), mediating chemotherapy, radiotherapy and immunotherapy resistance. This review has highlighted how hypoxia regulates multiple pro-tumorigenic pathways through HIF to induce changes in the ECM, activate CAFs to enhance tumorigenesis and promote an immunosuppressive microenvironment. Additionally, the role of hypoxia in inducing changes to tumour cell metabolism was also discussed showing its role in resistance to therapies due to acidification of the TME (83).

Whilst there is strong evidence supporting the role of hypoxia in enhancing tumorigenesis and its role in complicating treatment in a clinical setting, there are still gaps in knowledge. It is important to be able to decipher the complexity of the TME including clarifying the contradictions in ECM data, and how hypoxia interacts with CAFs and their specific influence on both the ECM and TME. Many *in vitro* models used to study hypoxia often rely on 2D models of a single cell type which do not take into consideration the whole TME (367). Therefore, using new models able to recapitulate the hypoxic TME in 3D with multiple cell types may drive research further. This can provide novel insights into how the mechanosensory interactions influence resistance to therapies for example through matrix stiffness, and the specific cell types that can be targeted to evade this mechanism of resistance (354, 368). Representation of cycling hypoxia which is more physiologically relevant than acute or chronic hypoxia may be beneficial (3). Understanding the specific roles of CAFs within the TME is critical as there are multiple subtypes with differing roles. Until this is fully clarified, it will be difficult to develop targeted strategies

Further elucidation of the role of HIF-3 may provide important insights into HIF biology, and new mechanisms of therapeutic targets (369). Additionally, continued research into pseudohypoxia could provide a novel strategy to target tumours.

Hypoxia research has enabled the development of hypoxia-specific treatments that have shown some clinical success (97, 323, 362). As summarised by Hoskin (365), the problem lies with the lack of clinical implementation despite significant evidence supporting benefits of hypoxia modification. Development of hypoxic biomarkers is still of interest and perhaps indicates that there is a future for hypoxia-targeted therapies in clinics with better methods of stratifying patients. Some limitations with current biomarkers that have been developed is that they use platforms that are not clinically applicable. Furthermore, many of the biomarkers are not validated in prospective clinical trials. Understanding the best format for analysing hypoxia gene/protein signatures in tissue (fresh frozen/FFPE), and using platforms that are clinically available and affordable could help overcome this.

The complex hypoxic TME provides an opportunity for identifying novel therapeutic targets. However, as we have learnt from treatments already implemented in clinic, for example anti-PD-1 therapies, there are often mechanisms that induce resistance (351). Understanding the complex interactions between the different cell types within the hypoxic TME, and the differing expression patterns regulated by hypoxia could help develop more targeted treatments. Developing biomarkers using platforms that we know are clinically accessible can overcome the challenge of clinical implementation.

## Author contributions

KB: Writing – original draft, Writing – review & editing. CQ: Writing – review & editing. SL: Writing – review & editing. DS: Writing – review & editing. MK: Writing – review & editing. ET: Writing – review & editing. CW: Writing – review & editing. PH: Writing – review & editing. AC: Writing – review & editing.
